# Influenza-Activated ILC1s Contribute to Antiviral Immunity Partially Influenced by Differential GITR Expression

**DOI:** 10.3389/fimmu.2018.00505

**Published:** 2018-03-22

**Authors:** Neha Vashist, Stephanie Trittel, Thomas Ebensen, Benedict J. Chambers, Carlos A. Guzmán, Peggy Riese

**Affiliations:** ^1^Department of Vaccinology and Applied Microbiology, Helmholtz Centre for Infection Research, Braunschweig, Germany; ^2^Department of Medicine, Center for Infectious Medicine, Karolinska Institute, Karolinska University Hospital Huddinge, Stockholm, Sweden

**Keywords:** influenza, innate lymphoid cell 1, regulation, glucocorticoid-induced TNFR-related protein, cross-talk

## Abstract

Innate lymphoid cells (ILCs) represent diversified subsets of effector cells as well as immune regulators of mucosal immunity and are classified into group 1 ILCs, group 2 ILCs, and group 3 ILCs. Group 1 ILCs encompass natural killer (NK) cells and non-NK ILCs (ILC1s) and mediate their functionality *via* the rapid production of IFN-γ and TNF-α. The current knowledge of ILC1s mainly associates them to inflammatory processes. Much less is known about their regulation during infection and their capacity to interact with cells of the adaptive immune system. The present study dissected the role of ILC1s during early influenza A virus infection, thereby revealing their impact on the antiviral response. Exploiting *in vitro* and *in vivo* H1N1 infection systems, a cross-talk of ILC1s with cells of the innate and the adaptive immunity was demonstrated, which contributes to anti-influenza immunity. A novel association of ILC1 functionality and the expression of the glucocorticoid-induced TNFR-related protein (GITR) was observed, which hints toward a so far undescribed role of GITR in regulating ILC1 responsiveness. Overexpression of GITR inhibits IFN-γ production by ILC1s, whereas partial reduction of GITR expression can reverse this effect, thereby regulating ILC1 functionality. These new insights into ILC1 biology define potential intervention targets to modulate the functional properties of ILC1s, thus contributing toward the development of new immune interventions against influenza.

## Introduction

Innate lymphoid cells (ILCs) represent an expanding family of tissue resident innate effectors and are mainly enriched at mucosal barriers. They are classified into group 1 [natural killer (NK) cells and non-NK ILC1s], group 2 (ILC2s), and group 3 ILCs (ILC3s) based on their different transcription factors and cytokine secretion profiles (1). Group 1 ILCs consist of NK cells and ILC1s, express the transcription factor T-bet and promptly produce IFN-γ in response to activation by interleukin (IL)-12 and IL-18. These ILC1s share several phenotypic markers with NK cells. However, ILC1s also display significant differences to NK cells in terms of development, tissue distribution and functionality (2). In this context, ILC1s lack Eomes expression unlike NK cells, which express both T-bet and Eomes (3). In contrast to NK cells, ILC1s also do not exhibit granzyme-mediated cytotoxicity but are capable of receptor-mediated killing by expressing the TNF-related apoptosis-inducing ligand (TRAIL) (4). Furthermore, NK cells play a critical role in the defense against viral infections, whereas ILC1s were reported to be crucial for protection against parasitic and bacterial infections at mucosal barriers (5–7). Activated NK cells can support protection against pathogens by initiating adaptive immunity next to cytotoxic responses. For example, a cross-talk between NK cells and dendritic cells (DCs) in the course of influenza infection was reported, whereby NK cell-derived IFN-γ resulted in DC activation and subsequent priming of CD8 T cells (8). Similar to NK cells, ILC2s and ILC3s were shown to communicate with antigen-presenting cells (APCs) thereby resulting in the priming of adaptive immune responses. In this regard, previous reports demonstrated that both ILC2s and ILC3s are capable to interact with adaptive immune cells (9). In this line, IL-13 produced by ILC2s was demonstrated to induce DC migration from lungs to draining lymph nodes (dLNs) which resulted in the priming of naive T cells (10). Likewise, GM-CSF production in the gut by ILC3s led to the secretion of TGF-β and IL-10 by macrophages and DCs subsequently leading to the proliferation of regulatory T cells. However, the contribution of ILC1s to the initiation of adaptive immunity is less well characterized as compared to other ILC subpopulations (2).

Infection with influenza A virus (IAV) can cause major epidemics or pandemics and despite the availability of vaccines, influenza is still responsible for 250,000–500,000 annual deaths (11). IAV infections are thus a major public health concern. Innate immunity represents a first line of defense in the combat against IAV infections. Studies in murine models revealed that NK cells utilize their cytotoxic functions to lyse influenza infected cells as well as initiate specific adaptive cytotoxic immune responses (8). Recent studies also demonstrated a role for ILC2 and ILC3 cells in the course of influenza infections (12–14). However, the contribution of ILC1s during IAV infection is still an open area of research (15).

The glucocorticoid-induced TNFR-related protein (GITR) is a receptor of the TNFR superfamily expressed on immune cell types such as regulatory T cells, naive T cells, and NK cells, as well as at lower levels on APCs (16). The ligand for GITR (GITR-L) is expressed on APCs, endothelial cells and activated T cells (17). GITR ligation supports the costimulation of CD4 and CD8 T cells, resulting in enhanced proliferation that in turn increases effector functions (18). GITR-GITR-L signaling is also involved in immune cell regulation. Thus, it can actively suppress regulatory T cells, thereby augmenting effector T cell responses (18, 19). Interestingly, a GITR-GITR-L interaction was also reported for NK cells, leading to enhanced NK cell cytotoxicity and functionality (20). Hence, stimulation *via* GITR engagement represents a mechanism connected to activation as well as regulation of both innate and adaptive immune cells. Interestingly, GITR was shown to be important for CD8 T cell functionality and subsequently the survival of mice following severe influenza infection (21).

In this study, the impact of ILC1s during infection with the IAV H1N1 was investigated, as well as a potential mechanism involved in ILC1 activation and regulation. The obtained results highlight the role played by ILC1s in the course of IAV infection partly mediated by the cross-talk with cells of the innate and adaptive immune system crucial for clearing IAV infection. Furthermore, the performed studies identified the GITR signaling pathway as a potential mechanism modulating ILC1 functionality.

## Materials and Methods

### Mice

C57BL/6 (H-2^b^) female mice aged 6–8 weeks were purchased from Harlan Winkelmann GmbH (Borchen, Germany). RAG2^−/−^ and RAG2^−/−γ^c^−/−^ mice (C57BL/6 background) were bred at the animal facility of the Helmholtz Centre for Infection Research, Braunschweig. Mice were treated in consensus with local and European Community guidelines and were housed under specific pathogen-free conditions in individual ventilated cages with food and water *ad libitum*. The performed animal experiments were approved by the local government in Braunschweig, Germany (AZ: 33.42502-04-13/1281 and AZ: 33.19-42502-04-16/2280). All animal experimentation were performed under both institutional and national guidelines.

### Virus Strains and *In Vivo* Infection

Mouse-adapted influenza A/PR/8/34 (H1N1 PR8) was provided by Dr. Paulina Blazejewska and Dr. Klaus Schughart (Helmholtz Centre for Infection Research). The recombinant influenza A/PR/8/34 strains which either contain the OVA epitope SIINFEKL (OT-I PR8) or the OVA epitope aa323-aa393 (OT-II PR8) were provided by Dr. David Topham (University of Rochester Medical Center) and Dr. Stephen Turner (The Peter Doherty Institute for Infection and Immunity Department of Microbiology and Immunology), respectively. The virus was propagated in the chorioallantoic fluid of 10-days-old pathogen free embryonated chicken eggs at 37°C, aliquoted and stored at −80°C as previously described (22). IAV infection was performed with a sub-lethal dose. To this end, female mice were anesthetized intraperitoneally (i.p.) with a 100 µl mixture of ketamine (100 mg/kg, 10% WDT eG, Germany) and Xylavet (20 mg/kg, cp Pharma, Germany) in NaCl (0.9% BRAUN, Germany) and administered intranasally (i.n.) with a total volume of 20 µl comprising of sterile PBS and 2 × 10^3^ foci forming units (ffu) of H1N1 PR8. To assess viral infectivity and viral titers post influenza infection, a foci assay was performed with homogenized lung samples as previously described (22). Briefly, Madin-Darby canine kidney cells were incubated with the lung homogenate and subsequently stained for the influenza nucleocapsid to detect foci [primary antibody; anti-influenza nucleocapsid (NP) polyclonal goat antibody, ViroStat, USA and secondary antibody; antigoat-HRP, KPL, USA]. Viral titers were calculated as ffu per ml of infectious homogenate.

### Preparation of Single Cell Suspensions

Lungs, spleens, and dLNs (cervical and mediastinal) were removed from euthanized mice. Broncheoalveolar lavage (BAL) samples were collected by two intratracheal washes with 1 ml 5% FCS PBS. To isolate lung-derived lymphocytes, lungs were mashed in a 100 µm nylon strainer and digested with 0.2 mg/ml collagenase D (Roche, Germany) and 20 µg/ml DNase I (Roche, Germany) in 5% FCS RPMI 1640 (Life technologies, UK). Density gradient centrifugation with Easycoll (Biochrome GmbH, Germany) was then utilized to segregate single cell suspensions from the enzyme-digested lung tissue. To generate single cell suspensions from spleens and dLNs, the organs were mashed through 100 µm nylon cell strainers. Splenic erythrocytes were destroyed with ammonium chloride potassium (ACK) lysis buffer. Lung lymphocytes and splenocytes derived from the *in vivo* infection experiments were incubated with medium containing brefeldin A (5 µg/ml) and monensin (6 µg/ml) for 3 h at 37°C. Following, the single cell suspensions were used for flow cytometry analysis.

### IL-12 and IL-18 Detection Post-IAV Infection

The changes in the cytokine levels of IL-12 and IL-18 in the BAL and sera of H1N1-infected mice were analyzed using a bead-based flow cytometry approach (Affymetrix/eBioscience) according to the protocol provided by the supplier. Samples were acquired using the FACS Fortessa (BD Bioscience, USA) and data analysis was performed with FlowCytomix™ Pro 3.0 software (Affymetrix/eBioscience).

### *In Vitro* Generation of ILC1s and Cytokine Stimulation

Single cells were obtained from RAG2^−/−^ bone marrows as described previously (23) and cultured for 4 days with murine stem cell factor and FLT-3 ligand (100 ng/ml, Peprotech, USA) in RPMI complete media [supplemented with 10% FCS (Sigma-Aldrich, Germany), 1 mM sodium pyruvate (Sigma, UK), 10 mM Hepes and 1 mM non-essential amino acids (Hyclone, Thermo-Scientific, USA), 20 µM 2-β mercaptoethanol, and 50 µg/ml gentamycin (Gibco, Life Technologies, USA)]. At day 5 of the culture, CD90.2^+^ cells were positively selected using magnetic microbeads (Miltenyi Biotech, Germany) and cultured with IL-7 and IL-15 for further 2 days (100 ng/ml, Peprotech, USA). Subsequently, CD127^+^ cells were isolated by positive selection using biotin-conjugated anti-CD127 and anti-biotin magnetic microbeads (Miltenyi Biotech, Germany) and cultured for further 4 days with IL-7 and IL-15. The obtained ILC1 population was assessed by flow cytometry and either used for coculture studies to address the cross-talk with bone marrow-derived dendritic cells (BMDCs) and T cell immune populations or for IL-12 and IL-18 (100 ng/ml, Peprotech, USA/MBL, USA) cytokine stimulation to assess the immune profile of activated ILC1s.

### BMDC Generation and *In Vitro* Infection

Flt-3 ligand-derived BMDCs were generated as described previously (24). Briefly, BM cells were stimulated with 100 ng/ml of FLT-3 ligand (Peprotech, USA) for 7–8 days in RPMI complete medium. BMDCs were subsequently stained with CFSE (carboxyfluorescein succinimidyl ester) for 15 min at room temperature in 0.5% FCS-PBS. Afterward, the obtained BMDCs were infected for 1 h with the mouse-adapted influenza A/PR/8/34, OT-I PR8, or OT-II PR8 strains with a multiplicity of infection (MOI) of 1 and further cultured for other 5 h. Then, BMDCs were harvested and stained with a monoclonal antibody against IAV nucleoprotein (NP; 431, FITC Anti-NP, Abcam) to confirm infection.

### Coculture Experiments

To assess the cross-talk of ILC1s with infected BMDCs and CD8/CD4 T cells, *in vitro* coculture studies were performed overnight. To this end, ≈7.5 × 10^4^ CFSE labeled BMDCs infected with H1N1 PR8 were cocultured with ILC1s at a ratio of 1:1. Furthermore, ≈4.5 × 10^4^ CFSE-labeled BMDCs infected with OT-II PR8 or OT-I PR8 were cocultured at a ratio of 1:1:1 with ILC1s and CD4 or CD8 T cells isolated by negative selection from splenocytes of OT-II (93% purity) and OT-I (92.5% purity) mice, respectively, utilizing the corresponding isolation kits (Miltenyi Biotech, Germany). Brefeldin A (5 µg/ml) and monensin (6 µg/ml) were added for the last 3 h of coculture to prevent protein export. Flow cytometry staining was performed to assess changes in ILC1, BMDC, and CD4/CD8 phenotype and functionality.

### Adoptive Transfer Experiments

To address the *in vivo* impact of ILC1s in the course of H1N1 infection, 2 × 10^5^ *in vitro*-generated ILC1s diluted in 100 µl PBS were adoptively transferred i.v. into RAG2^−/−^γc^−/−^ mice. To study whether adding adaptive immune cells to ILC1s affects the viral load, CD4 and CD8 T cells were isolated from OT-II- or OT-I-derived splenocytes, respectively. Then, 10^5^ CD4 or CD8 T cells were adoptively transferred i.v. into RAG2^−/−^γc^−/−^ mice together with *in vitro*-generated ILC1s at a 1:1 ratio. Influenza infection was performed 24 h after adoptive transfer.

### *In Vivo* CD8 and CD4 T Cell Depletion

Monoclonal anti-mouse CD8 (YTS 169.4, BioXCell, USA) and CD4 antibodies (GK1.5, BioXCell, USA) were used to deplete CD8 and CD4 T cells *in vivo*, respectively, at 0 and 3 days post infection (dpi) (200 µg/animal in 200 µl PBS, i.p.). The depletion was confirmed by flow cytometry analysis of blood samples collected *via* the retro-orbital vein 2 and 5 dpi using antibodies against CD8 (53-6.7, FITC, BD Biosciences) and CD4 (RM4-5, PE-Cy7, eBioscience).

### *In Vitro* and *In Vivo* Targeting of GITR/GITR-L

The GITR-Fc chimera fusion protein (10 µg/ml, R&D Systems, USA) was used to manipulate GITR expression *in vitro* according to published reports (25). No intrinsic signaling-induced activation on GITR-insufficient splenocytes was observed (Figure S7 in Supplementary Material). The GITR targeting was performed overnight for the BMDC-ILC1 coculture system. Fluorescence minus one was used as negative control. For *in vivo* targeting of GITR-GITR-L interactions, *Invivo*MAb anti mouse GITR (DTA-1, BioXCell, USA)/Isotype (LTF-2, BioXCell, USA), was used to stimulate GITR signaling (500 µg/animal in 200 µl PBS, i.p.). The GITR-Fc chimera fusion protein (6.25 µg/animal in 200 µl PBS) was administered i.p. *in vivo* as a GITR-L agonist. For both *in vivo* targeting strategies, H1N1 infection was performed 1 day post treatment (2 × 10^3^ ffu/animal in 20 µl, i.n.).

### Flow Cytometry Analysis

Single cell suspensions from *in vivo* experiments and *in vitro* cocultures were incubated with Fc-block followed by surface marker staining. Subsequently, cells were fixed and permeabilized for intracellular and intranuclear staining with the provided solutions according to the manufacturer’s protocol (BD Bioscience, USA/Foxp3 staining kit, eBioscience, USA). FACS LSR II and Fortessa (BD Bioscience, USA) were used to acquire stained samples and the analysis was performed using FlowJo (TreeStar Inc.). The following antibodies were utilized to perform flow cytometry staining: LIVE/DEAD Fixable Blue Dead cell stain kit (UV excitation, Invitrogen, USA), CD90.2 (53.2-1, BV785, Biolegend), CD127 (A7R34, PE-Cy5/biotin, Biolegend), NKp46 (29A1.4, A700, BD Bioscience/19A1.4, APC, eBioscience), CD3/CD19/Gr1/Ter-119 (17A2/6D5/RB6-8C5/TER-119, BV421, Biolegend), TRAIL (N2B2, biotin–streptavidin BV650, Biolegend), GITR (YGITR765, PE, Biolegend), CD28 (37.51, PerCP-Cy5.5, Biolegend), CTLA-4 (UC10-489, BV605, Biolegend), CD49a (Ha31/8, BV510, BD Bioscience), CD11c (N418, PE-Cy7, Biolegend), CD11b (M1/70, BV605, Biolegend), B220 (RA3-6B2, PE-Cy5, Biolegend), CD103 (2E7, Alexa 488, Biolegend), F4/80 (BM8, Percpef710, Biolegend), CD80 (16-10A1, BV421, Biolegend), CD86 (GL-1, BV650, Biolegend), CD40 (HM40-3, Alexa 647, Biolegend), MHC cl. II (M5/114.15.2, A700, Biolegend), GITR-L (YGL 386, PE, Biolegend), CD3 (17A2, BV785, Biolegend), CD8 (53-6.7, APCef780, eBioscience), CD4 (RMA-4, APC, BD Biosciences), IFN-γ (XMG1.2, BV711, Biolegend), TNF-α (MP6-XT22, PE-Dazzle, Biolegend), IL-2 (JES6-5H4, APC-Cy7, BD Bioscience), T-bet (4B10, PE-Cy7, Biolegend), Eomes (Dan11mag, FITC, eBioscience), RORγt (AFKJS-9, APC, eBioscience), and CD16/CD32 (2.4G2, Fc block, BD Biosciences).

### Statistical Analysis

The obtained data were statistically analyzed using GraphPad Prism 6.0 (GraphPad Software, USA). Statistical differences were assessed by non-parametric [Mann–Whitney’s or Kruskal–Wallis (with Dunns’ posttest) or parametric tests (Student’s *t*-test or One-way ANOVA)]. Values of *p* ≤ 0.05 were recognized as statistically significant.

## Results

### H1N1 Infection Leads to Functional Activation of ILC1s

**Figure 1:**
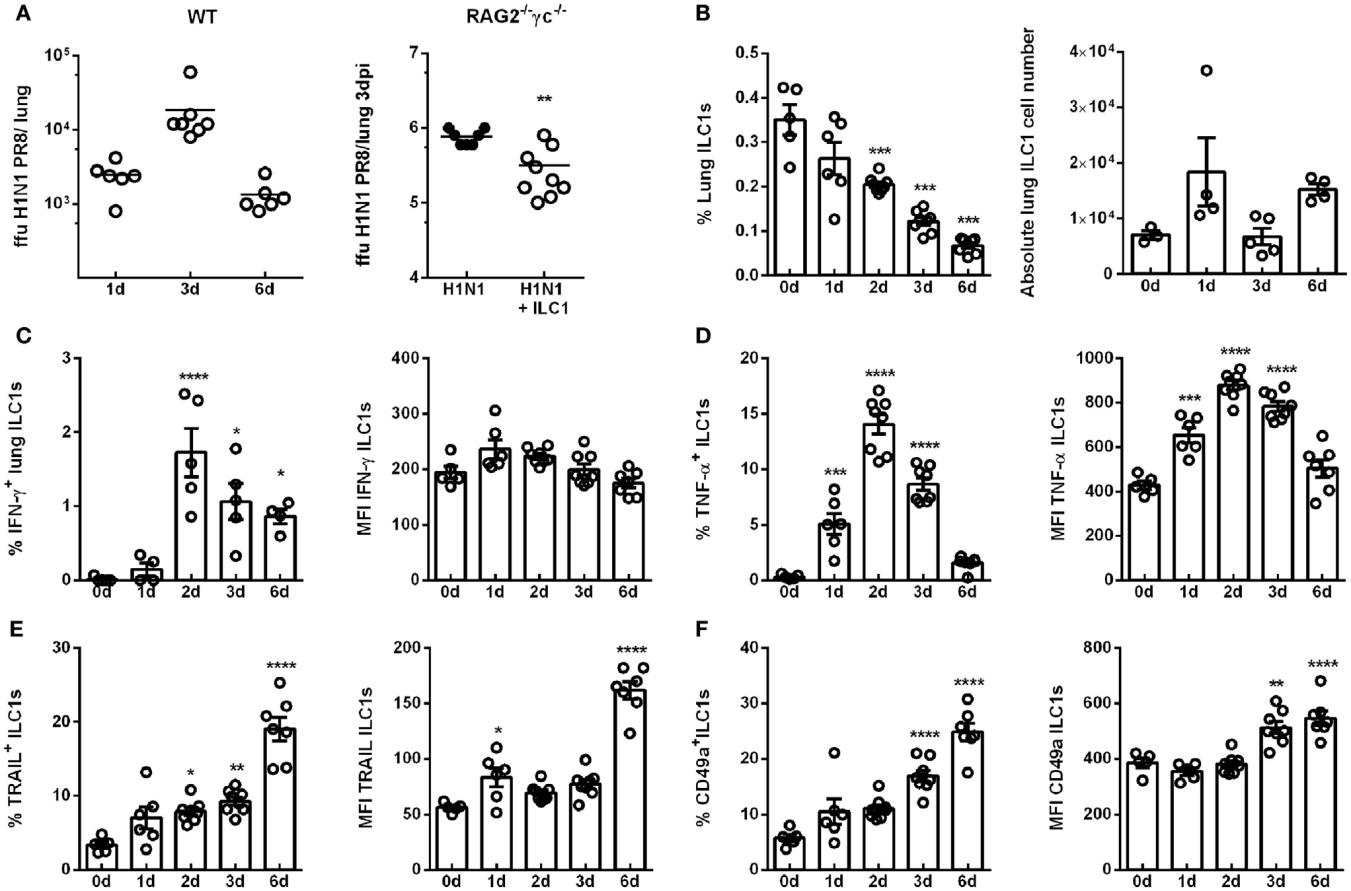
H1N1 infection leads to enhanced ILC1 activation and functionality. Wild-type mice and RAG2^−/−^γc^−/−^ mice were infected i.n. with 2 × 10^3^ ffu of the H1N1 PR8 strain. **(A)** Scatter plots represent viral loads of wild-type mice (left panel) and RAG2^−/−^γc^−/−^ mice adoptively transferred with 2 × 10^5^ *in vitro*-generated ILC1s/animal (right panel; *n* = 4–8) as determined by foci assay and depicted as ffu per lung at the indicated time points. Lymphocytes derived *ex vivo* from lungs of infected wild-type mice were incubated at 37°C in medium containing brefeldin and monensin for 3 h prior to flow cytometry staining of markers related to ILC1 activation and functionality. **(B)** Frequencies and absolute numbers of lung-derived ILC1s. Frequencies and mean fluorescence intensities (MFI) of **(C)** IFN-γ, **(D)** TNF-α, **(E)** TRAIL, and **(F)** CD49a expressing lung ILC1s. Shown is one out of three independent experiments (*n* = 4–8). Bars with scatter plots represent MFI and range in frequencies with the horizontal line drawn at the mean. Asterisks denote significant values as calculated by nonparametric Mann–Whitney’s test (viral loads) or One-way ANOVA (frequency and MFI) as compared to uninfected samples; ****p ≤ 0.0001; ***p ≤ 0.001; **p ≤ 0.01; *p ≤ 0.05.

To address the role of ILC1s in mucosal-transmitted influenza infection, wild-type mice were infected i.n. with a sublethal dose of the H1N1 IAV strain (A/PR/8/34) and the body weight was monitored daily (Figure S2A in Supplementary Material). The virus titers in the lungs of infected mice evaluated at different time points peaked dpi and decreased 6 dpi (Figure 1A). To assess the direct contribution of ILC1s during the course of IAV infection, *in vitro*-generated ILC1s (generation protocol and phenotypic characterization; Figures S1A–C in Supplementary Material) were adoptively transferred into RAG2^−/−^γc^−/−^ mice followed by subsequent H1N1 PR8 infection. The weight loss upon infection did not differ in ILC1-reconstituted and ILC1-insufficient mice (Figure S2B in Supplementary Material). The lung viral burden was significantly reduced, although not very remarkably, 3 dpi in the ILC1-competent mice as compared to mice lacking ILC1s (Figure 1A). This effect was not observed 6 dpi (Figure S2B in Supplementary Material).

Next, the impact of influenza infection on the ILC1 phenotype and functionality was investigated in wild-type mice. To this end, lung-derived ILC1s were analyzed by flow cytometry for their frequency and the expression of markers related to activation and functionality (gating strategy for the identification of ILC1s; Figure S3 in Supplementary Material). The frequencies of lung ILC1s were significantly decreased 2, 3, and 6 dpi, however, the absolute numbers of lung ILC1s were not significantly affected (Figure 1B). Proinflammatory cytokines play an important role in the course of viral infections, being involved in both viral clearance and immune pathology. Thus, changes in the cytokine profile (secretion of IFN-γ and TNF-α) of ILC1s in the course of IAV infection were addressed (Figure S4A in Supplementary Material). Interestingly, increased frequencies of IFN-γ-secreting lung ILC1s were observed 2 and 3 dpi, whereas no significant alterations were detected in the expression density (i.e., MFI; Figure 1C). Furthermore, significantly elevated frequencies and expression densities of lung-derived TNF-α-producing ILC1s were detected 1, 2, and 3 dpi (Figure 1D). The analysis of surface markers which depict the activation status of lung ILC1s also revealed significantly increased frequencies of TRAIL^+^ ILC1s 2 and 3 dpi, which were further enhanced 6 dpi (Figure 1E; Figure S4B in Supplementary Material). Significantly enhanced expression densities of TRAIL on lung ILC1s were observed 1 and 6 dpi. Furthermore, the frequencies as well as the expression densities of CD49a^+^ lung-derived ILC1s were significantly increased 3 and 6 dpi (Figure 1F; Figure S4B in Supplementary Material). To further phenotypically characterize the *ex vivo* derived lung ILC1s, the expression of the markers DNAM-1, CD69, CD28, CD62L, CD11b, Ly49E/F, KLRG1, and DX5 (Figures S5A,B in Supplementary Material) were analyzed on lungs ILC1s at steady state (0 day) and 3 dpi. Interestingly, lung ILC1s also displayed a significantly enhanced expression density and frequency of CD69^+^ ILC1s (Figure S5C in Supplementary Material).

The evaluation of splenic ILC1s revealed no changes in their frequency but a decrease in the absolute ILC1s counts upon H1N1 infection (Figure S4C in Supplementary Material). The expression density of IFN-γ on splenic ILC1s was not affected by infection, whereas an enhanced expression density of TNF-α was observed (Figures S4D,E in Supplementary Material). Furthermore, an increased frequency of splenic ILC1s expressing TRAIL was found (Figure S4F in Supplementary Material). In contrast to lung ILC1s, splenic ILC1s showed a significantly decreased expression of CD49a 6 dpi (Figure S4G in Supplementary Material).

To further assess the observation of activated ILC1s upon influenza infection, ILC1-insufficient and ILC1-reconstituted RAG2^−/−^γc^−/−^ mice were infected with H1N1 PR8. Lung-derived lymphocytes were subsequently analyzed for changes in functionality of adoptively transferred *in vitro*-expanded ILC1s (Figures S2C,D in Supplementary Material). T-bet^+^Eomes^−^ ILC1s were identified in ILC1-reconstituted uninfected and infected RAG2^−/−^γc^−/−^ mice. Although reconstituted ILC1s did not display functional activation with regard to the secretion of IFN-γ and TNF-α upon influenza infection at the investigated time points, increased expression densities of TRAIL and CD49a on ILC1s were detected (Figure S2D in Supplementary Material).

The cytokine milieu in the lung shapes the subsequent immune response to infection. It was reported that IL-12 and IL-18 can synergistically enhance IFN-γ production by NK cells, thereby enhancing their cytotoxic potential against influenza infection (26, 27). A cytometric bead array performed after H1N1 infection using BAL revealed increased levels of IL-12 and IL-18 3 dpi (Figure S4H in Supplementary Material). Thus, influenza-induced secretion of IL-12 and IL-18 might further contribute to ILC1 activation. These findings demonstrate enhanced activation and functionality of ILC1s at the level of the systemic and local compartments, thereby suggesting a role of ILC1s in the course of IAV infection.

### Enhanced GITR Expression Is Associated With ILC1 Activation

**Figure 2:**
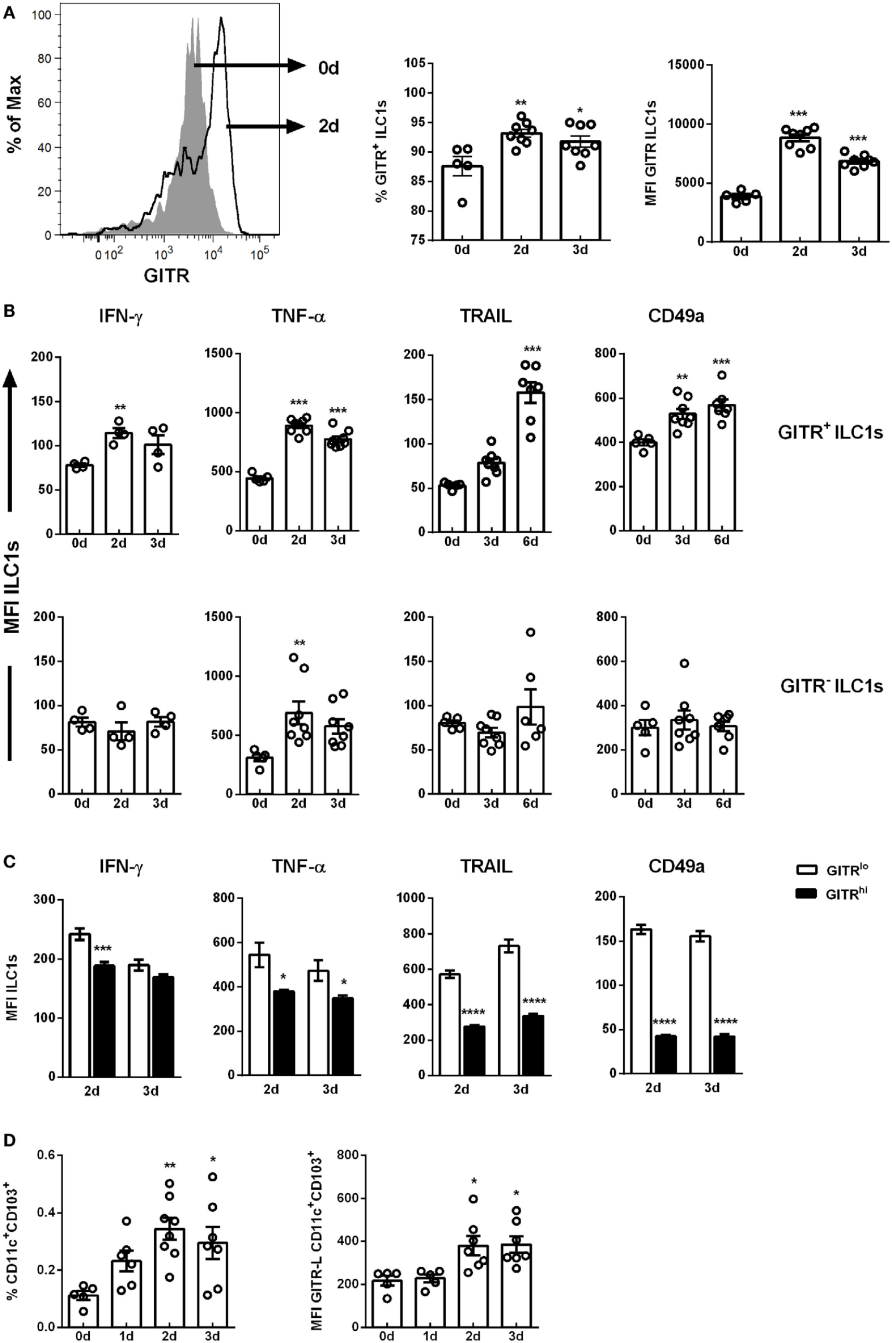
GITR expression defines ILC1 functionality. Wild-type mice were infected i.n. with 2 × 10^3^ ffu of the H1N1 PR8 strain. Lymphocytes derived *ex vivo* from lungs (for ILC1 profile) or dLNs (mediastinal and cervical, for antigen-presenting cell profile) of infected mice were incubated at 37°C in medium containing brefeldin and monensin for 3 h prior to the flow cytometry staining of markers related to ILC1 activation and functionality as well as DC markers, respectively (*n* = 5–8). **(A)** Representative histogram of GITR expression by ILC1s, GITR^+^ ILCs expressed as frequency of ILC1s and MFI of GITR-expressing ILC1s. **(B)** MFI of IFN-γ, TNF-α, TRAIL, and CD49a of GITR^+^ and GITR^−^ ILC1s. **(C)** MFI of IFN-γ, TNF-α, TRAIL, and CD49a of GITR^lo^ and GITR^hi^ ILC1s. **(D)** Frequencies of CD11c^+^CD103^+^ DCs and their expression densities of GITR-L. Bars with scatter plots represent MFI and range in frequencies with the horizontal line drawn at the mean. MFI and frequency data are from one out of two independent experiments. Asterisks denote significant values as calculated by One-way ANOVA as compared to uninfected samples; ****p ≤ 0.0001; ***p ≤ 0.001; **p ≤ 0.01; *p ≤ 0.05.

The dynamic changes observed in the number and functional properties of ILC1s during H1N1 PR8 infection raised the question of mechanisms regulating this process. In the course of viral infections, the interaction of GITR, either expressed by T cells or NK cells, and the corresponding ligands were reported to be associated with the activation of immune cells and their regulation (28). Thus, the H1N1 *in vivo* infection model of wild-type mice was utilized to address the potential role played by GITR for ILC1 regulation. H1N1 infection resulted in significantly increased frequencies of GITR^+^ ILC1s 2 and 3 dpi as well as elevated expression densities of GITR on ILC1s (Figure 2A). Splenic ILC1s displayed similar changes in their GITR expression density and frequency (Figure S6E in Supplementary Material).

To investigate whether GITR expression correlates with ILC1 functionality following H1N1 infection, GITR^+^ and GITR^−^ ILC1 subsets were analyzed for their potential to secrete cytokines. Although the overall expression density of IFN-γ was not significantly affected in the course of H1N1 infection (Figure 1D), GITR expression-dependent changes were detected. An enhanced IFN-γ expression density, up-regulation of the surface markers TRAIL and CD49a were observed on the GITR^+^ ILC1s post infection (Figure 2B; Figure S6D in Supplementary Material). In contrast, the GITR^−^ subset did not display any changes with regard to cytokine secretion or surface marker expression. Furthermore, GITR^+^ lung ILC1s displayed elevated expression density of the activation marker CD69 as compared to GITR^−^ lung ILC1s (fold change: 6.195 vs. 2.30) (Figure S5D in Supplementary Material). An increased TNF-α production was detected independent of GITR expression (Figure 2B). The analysis of splenic ILC1s showed a similar pattern, with a GITR-independent secretion of TNF-α, and increased expression of TRAIL in GITR^+^ ILC1s (Figure S6F in Supplementary Material). However, no changes were observed with regard to the secretion of IFN-γ (data not shown). A decreased expression of CD49a was mainly observed on the GITR^+^ ILC1 subset (Figure S6F in Supplementary Material).

Interestingly, GITR^+^ lung-derived ILC1s could be further differentiated into GITR^lo^ and GITR^hi^ ILC1s 2 and 3 dpi (Figure S6B in Supplementary Material). The functional comparison of GITR^lo^ and GITR^hi^ ILC1s revealed that GITR^lo^ ILC1s represent the more functional subset as compared to GITR^hi^ ILC1s. The expression densities of IFN-γ, TNF-α, TRAIL and CD49a on the GITR^hi^ ILC1s were significantly lower as compared to GITR^lo^ ILC1s 2 and 3 dpi (Figure 2C). Further studies were performed to assess the impact of H1N1 infection on the GITR-L expression. To this end, migratory DCs from dLNs (mediastinal and cervical) were analyzed to determine changes in their frequencies and GITR-L expression. The analysis revealed elevated frequencies of migratory CD11c^+^CD103^+^ DCs 2 and 3 dpi and increased expression densities of the GITR-L (Figure 2D). The obtained data suggest that H1N1 infection results in enhanced expression of GITR on lung ILC1s and GITR-L on CD11c^+^CD103^+^ DCs, which in turn is associated with an improved activation status and augmented IFN-γ production by ILC1s. However, overexpression of GITR can be associated with a more exhausted functional profile of ILC1s.

### GITR Expression Defines Responsiveness of ILC1s to Cytokine Stimulation

**Figure 3:**
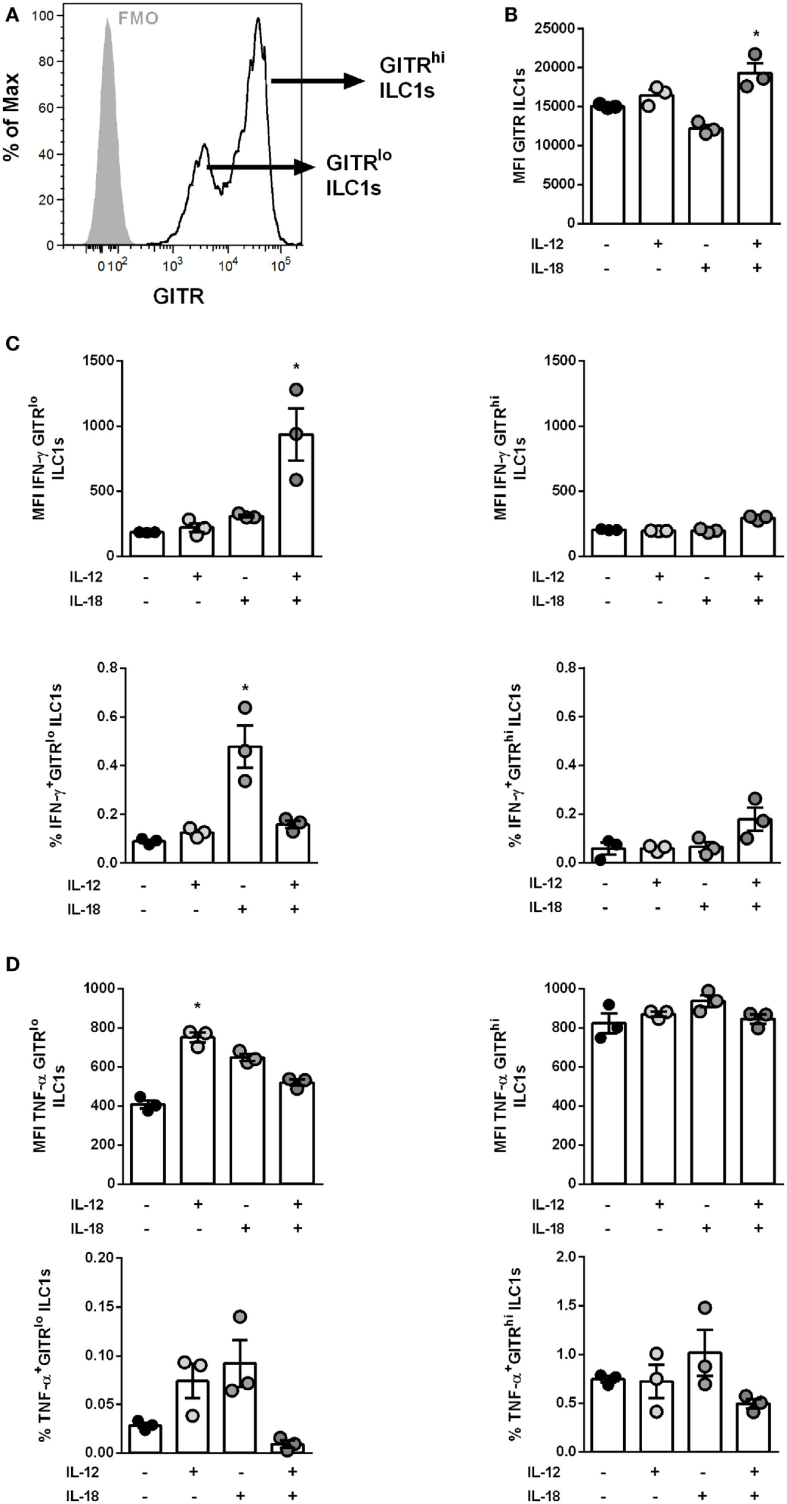
GITR expression level defines responsiveness of IL-12- and IL-18-induced ILC1 activation. *In vitro*-generated ILC1s were stimulated with 100 ng/ml of IL-12 ± IL-18 for 48 h and incubated at 37°C in medium containing brefeldin and monensin for 3 h prior to the flow cytometry of markers related to ILC1 activation and functionality. The bars with scatter plots represent the MFI values (*n* = 3–4). **(A)** Representative histogram depicts GITR expression on ILC1s at steady state (gray filled line = fmo control, black line = ILC1s) and **(B)** MFI of GITR expression by ILC1s post-IL-12 ± IL-18 treatment. Changes in expression densities and frequencies of GITR^lo^ and GITR^hi^ ILC1s expressing **(C)** IFN-γ and **(D)** TNF-α. MFI data are representative from one out of two independent experiments. Asterisks denote significant values as calculated by nonparametric Kruskal–Wallis test (Dunn’s posttest) as compared to untreated samples; ****p ≤ 0.0001; ***p ≤ 0.001; **p ≤ 0.01; *p ≤ 0.05.

As already shown, increased levels of IL-12 and IL-18 were detected in the BAL of H1N1 PR8-infected mice (Figure S4H in Supplementary Material). In order to assess whether GITR expression impacts the functional status of ILC1s, *in vitro-*generated ILC1s were stimulated with IL-12 ± IL-18. Similar to the findings observed *in vivo, in vitro*-generated ILC1s displayed two subpopulations differently expressing GITR that were grouped into GITR^lo^ and GITR^hi^ (Figure 3A). Combined treatment with IL-12 and IL-18 resulted in significantly elevated GITR expression, whereas IL-12 or IL-18 alone did not induce significant changes (Figure 3B). Furthermore, ILC1s depicted enhanced cytokine production following IL-12 and IL-18 stimulation (Figure S6G in Supplementary Material). GITR expression was clearly associated with the activation status of *in vitro*-generated ILC1s. The assessment of the ILC1 cytokine profile after IL-12 ± IL-18 treatment showed that IFN-γ production was mainly enhanced in GITR^lo^ ILC1s (fold change: 5.07) but not GITR^hi^ ILC1s (fold change: 1.44) as compared to untreated controls (Figure 3C). Similarly, the frequency of IFN-γ secreting GITR^lo^ ILC1s but not GITR^hi^ ILC1s was increased as a result of IL-18 treatment as compared to untreated ILC1s (Figure 3C). The analysis of TNF-α secretion revealed that following IL-12 ± IL-18 treatment GITR^lo^ ILC1s expressed higher amounts of TNF-α, whereas no cytokine-induced increase was observed for GITR^hi^ ILC1s (Figure 3D). The frequency of TNF-α producing GITR^lo^ ILC1s displayed a similar activation pattern (Figure 3D). Thus, the correlation between GITR expression levels and the secretion of cytokines upon *in vitro* stimulation supports a potential role of GITR in the overall regulation of ILC1 activation and functionality.

### Bidirectional Interaction Between ILC1s and DCs in the Course of IAV H1N1 Infection

DCs represent a fundamental innate cell population for the generation of influenza-specific adaptive immunity. In the context of intracellular bacterial infections, ILC1s were described to communicate indirectly with DCs *via* DC-derived IL-12 and IL-1β (7). To address whether ILC1s cross-talk with DCs in the course of IAV infection, an *in vitro* H1N1 infection model with BMDCs was established.

**Figure 4:**
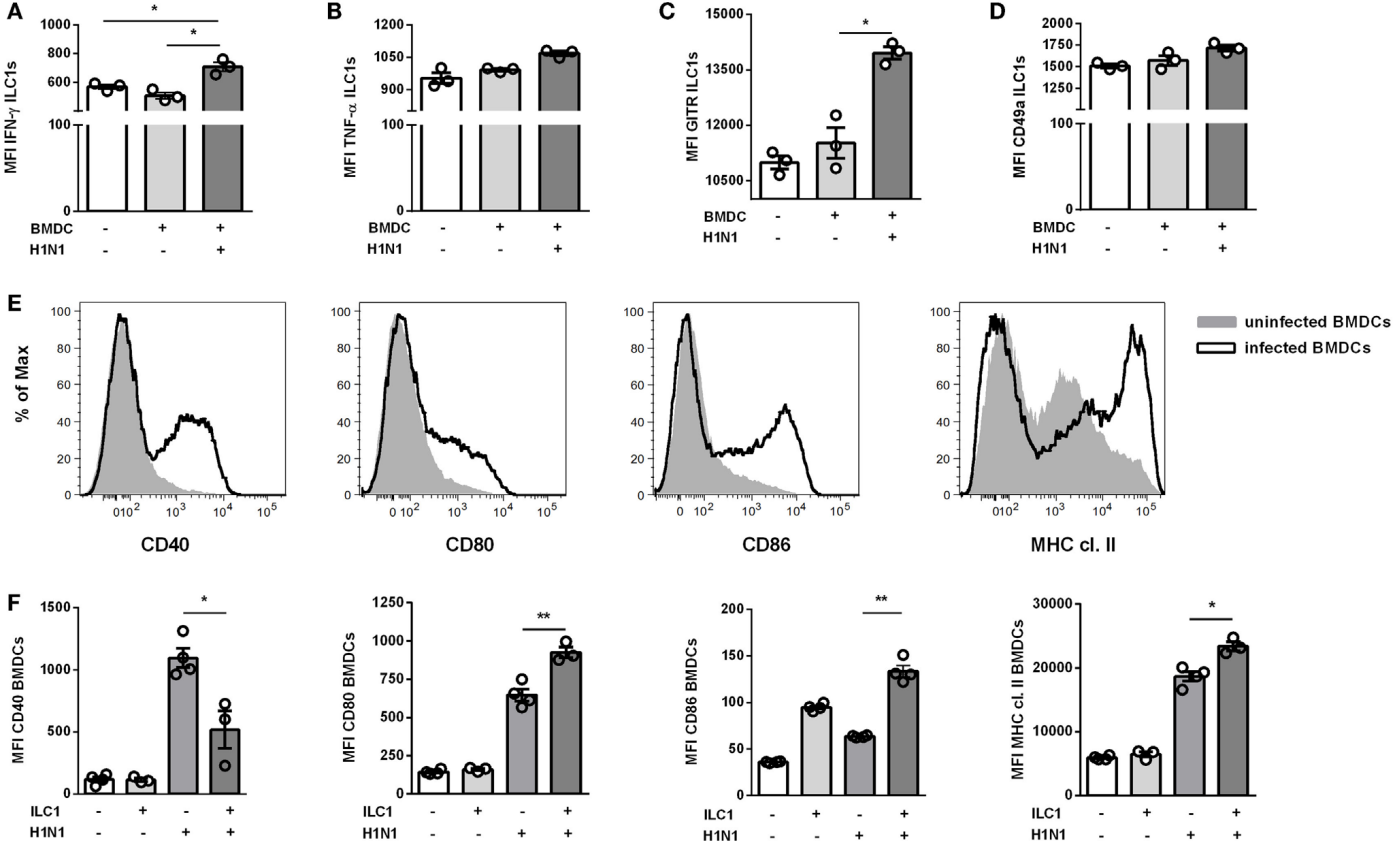
ILC1s are engaged in cross-talk with DCs during H1N1 infection *in vitro*. BMDCs generated from wild-type mice using the FLT-3 ligand stimulation were infected with the H1N1 PR8 strain (at a MOI of 1). *In vitro*-generated ILC1s cultured overnight with infected or uninfected BMDCs at a 1:1 ratio were stained for flow cytometry analysis after 3 h incubation in media with brefeldin and monensin. MFI of ILC1s expressing **(A)** IFN-γ, **(B)** TNF-α, **(C)** GITR, and **(D)** CD49a upon coculture with H1N1-infected or uninfected BMDCs. **(E)** Representative histograms for the expression of CD40, CD80, CD86, and MHC cl. II on infected BMDCs. **(F)** MFI of CD11c^+^ BMDCs expressing CD40, CD80, CD86, and MHC cl. II after coculture with ILC1s. Bars with scatter plots represent the mean ± SEM (*n* = 3–4) and MFI data are representative from one out of three independent experiments. Asterisks denote significant values as calculated by nonparametric Kruskal–Wallis test (Dunn’s posttest); ****p ≤ 0.0001; ***p ≤ 0.001; **p ≤ 0.01; *p ≤ 0.05.

BMDCs infected with a MOI of 1 displayed an infection rate of ≈8%, as evaluated by intracellular expression of NP (Figure S8A in Supplementary Material). The *in vitro*-generated ILC1s added to the cocultures did not get infected (data not shown). The ILC1 cytokine expression profiles showed enhanced secretion of IFN-γ but not TNF-α following coculture with IAV infected BMDCs (Figures 4A,B). Similar to the *in vivo* observations, increased GITR expression densities were detected on ILC1s cocultured with H1N1-infected BMDC as compared to ILC1s cocultured with uninfected BMDCs (Figure 4C). With regard to CD49a expression, the analysis of ILC1s cocultured with uninfected and infected BMDCs revealed only minor changes as compared to ILC1 controls (Figure 4D). The activation status of infected BMDCs was subsequently assessed by their expression levels of specific maturation markers (CD40, CD80, CD86, and MHC cl. II). As expected, H1N1 infection induced enhanced expression of CD40, CD80, CD86, and MHC cl. II on BMDCs (Figure 4E). H1N1-infected BMDCs cocultured with ILC1s showed additionally elevated expression densities of CD80, CD86 and MHC cl. II as compared to infected BMDCs cultured alone (Figure 4F). The expression of CD40 was significantly reduced when ILC1s were added to the culture. These *in vitro* results suggest a cross-talk between BMDCs and ILC1s during the course of IAV infection.

### GITR Expression Modulates Influenza-Mediated ILC1 Functionality

**Figure 5:**
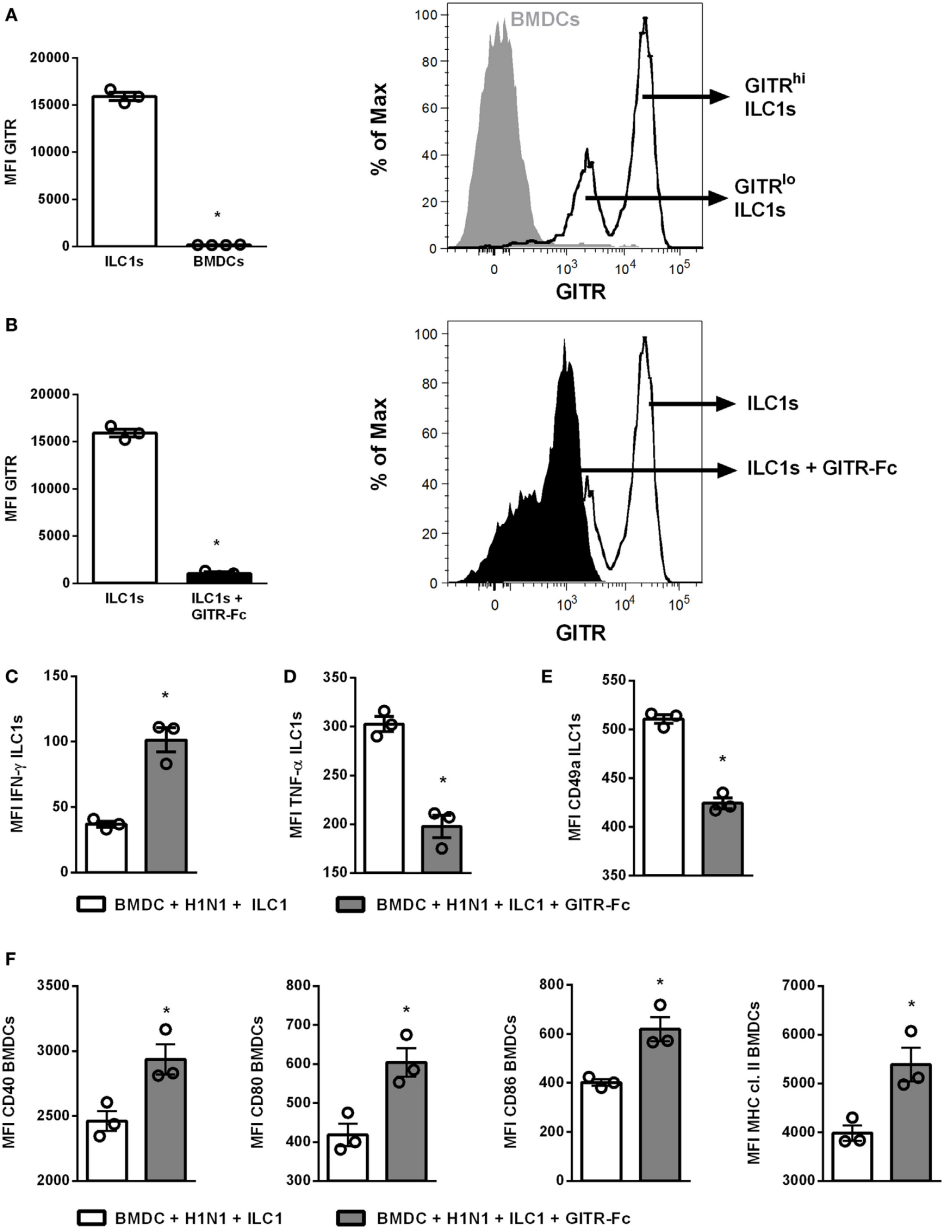
GITR-expression modulates ILC1 functionality upon coculture with H1N1-infected BMDCs. BMDCs generated from wild-type mice using FLT-3 ligand stimulation were infected with the H1N1 PR8 strain (at a MOI of 1). *In vitro*-generated ILC1s were cocultured overnight with infected or uninfected BMDCs at a 1:1 ratio. The recombinant mouse GITR-Fc chimera protein was applied to the coculture overnight to manipulate GITR expression. Surface and cytokine staining were performed for flow cytometry analysis after 3 h of incubation in media with brefeldin and monensin. **(A)** GITR expression by ILC1s and BMDCs represented as MFI and representative histogram. **(B)** MFI of GITR expression by ILC1s with and without GITR-Fc treatment and representative histogram. MFI of **(C)** IFN-γ, **(D)** TNF-α, and **(E)** CD49a expression after GITR-Fc treated ILC1s cocultured with H1N1-infected BMDCs. **(F)** MFI of CD40, CD80, CD86, and MHC cl. II expression by infected BMDCs post-GITR-Fc treatment. Bars with scatter plots represent the mean ± SEM (*n* = 3–4) and MFI data are representative from one out of two independent experiments. Asterisks denote significant values as calculated by nonparametric Mann–Whitney’s test; ****p ≤ 0.0001; ***p ≤ 0.001; **p ≤ 0.01; *p ≤ 0.05.

In order to further elucidate the impact of GITR on ILC1 functionality during H1N1 PR8 infection, studies manipulating the GITR expression were performed using the established *in vitro* H1N1 infection model of BMDCs and *in vitro*-generated ILC1s. Flow cytometry analysis revealed that GITR was mainly expressed by ILC1s but not by BMDCs (Figure 5A). Overnight treatment with a GITR-Fc protein resulted in a reduction of GITR expression on ILC1s (Figure 5B). The H1N1 infection BMDC-ILC1 coculture system revealed a significantly increased IFN-γ expression by ILC1s, accompanied by reduced expression of TNF-α and CD49a, in the presence of the GITR-Fc protein (Figures 5C–E). The reduced expression of GITR by ILC1s also affected the BMDC maturation. Enhanced expression of CD40, CD80, CD86, as well as MHC cl. II was observed upon adding the GITR-Fc protein (Figure 5F). The modulation of GITR expression by ILC1s in the coculture with uninfected BMDCs did not result in changes of the IFN-γ production by ILC1s or BMDC maturation, with the exception of CD80 expression (Figures S8B,C in Supplementary Material). Thus, the H1N1 infection BMDC-ILC1 coculture system suggests an influence of GITR signaling on the functional status of ILC1s and subsequently on DC activation and maturation.

### *In Vivo* Targeting of GITR Signaling Modulates Influenza-Mediated ILC1 Functionality

**Figure 6:**
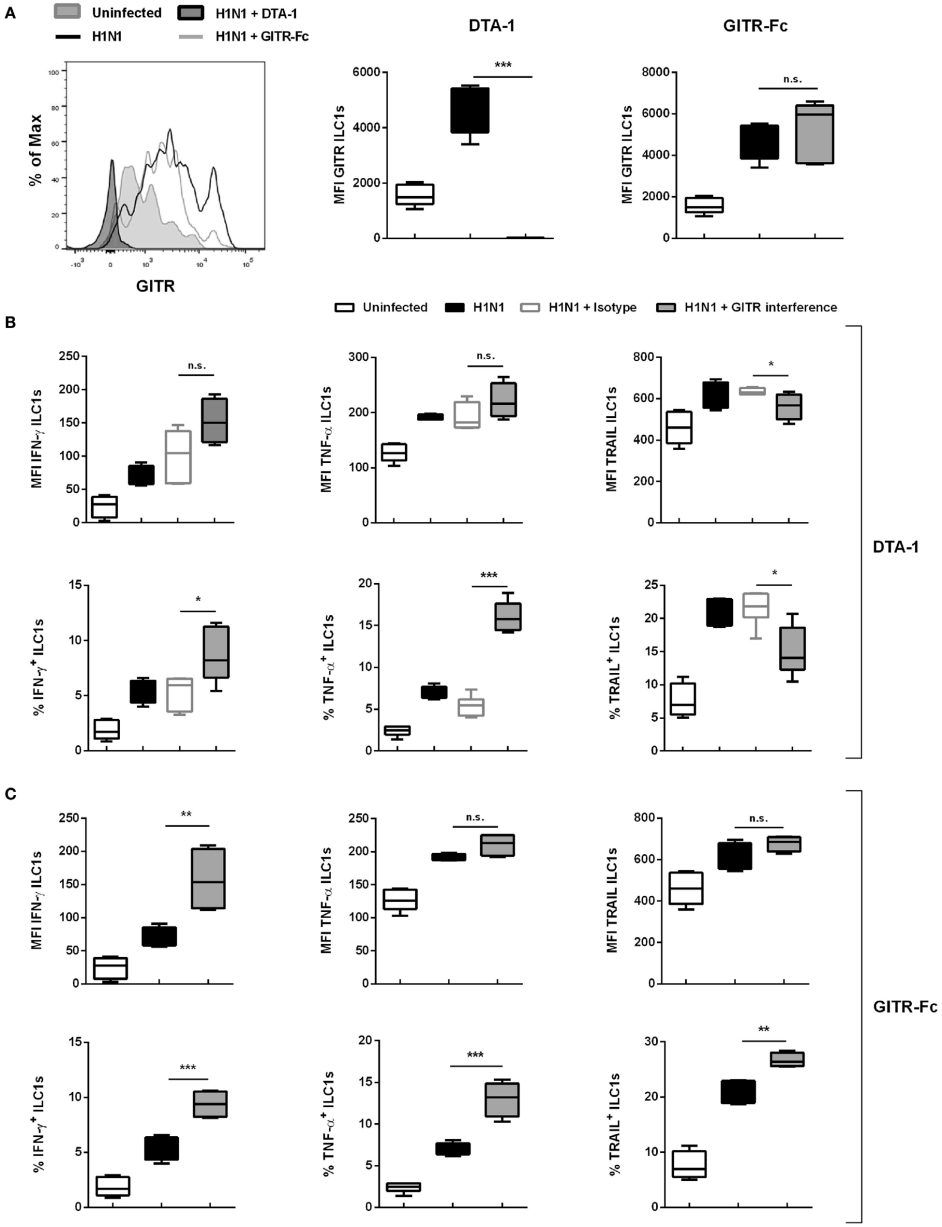
GITR–GITR-L interaction results in increased ILC1 functionality in the course of H1N1 infection. Wild-type mice were treated with either DTA-1 GITR agonist/isotype (500 µg/animal) or GITR-Fc fusion chimera protein (6.25 µg/animal) i.p. After 24 h mice were infected i.n. with 2 × 10^3^ ffu of the H1N1 PR8 strain. **(A)** Representative histograms and MFI of GITR expression by ILC1s. Lung-derived lymphocytes of infected wild-type mice 3 dpi were incubated at 37°C in medium containing brefeldin and monensin for 3 h prior to the flow cytometry staining with regard to markers related to ILC1 activation and functionality. **(B)** MFI and frequencies of lung-derived ILC1s expressing IFN-γ, TNF-α, and TRAIL from DTA-1-treated and control groups. **(C)** MFI and frequencies of lung-derived ILC1s expressing IFN-γ, TNF-α, and TRAIL from GITR-Fc-treated and control groups. Shown is one out of two independent experiments (*n* = 4–6). Box plots represent MFI and range in frequencies with the horizontal line drawn at the mean. Asterisks denote significant values as calculated by One-way ANOVA; ****p ≤ 0.0001; ***p ≤ 0.001; **p ≤ 0.01; *p ≤ 0.05; n.s., not significant.

To confirm the observed *in vitro* findings, the GITR signaling was targeted *in vivo*. For this purpose two approaches were utilized: Receptor triggering using an agonist (Anti-GITR DTA-1/DTA-1) or ligand activation with the GITR-Fc fusion protein. For this, wild-type mice were treated i.p. with the DTA-1 antibody or the GITR-Fc fusion protein. The mice were subsequently infected i.n. with a sublethal dose of the H1N1 strain PR8. Interestingly, viral load analysis revealed that DTA-1 treatment resulted in reduced lung viral titers 3 dpi, whereas no significant changes were observed upon GITR-Fc administration (Figure S8D in Supplementary Material). Lung-derived ILC1s were analyzed 3 dpi and evaluated for their functional changes by flow cytometry. The expression of GITR was significantly increased as a result of the influenza infection. Upon treatment with the DTA-1 antibody no GITR expression was detectable, whereas the treatment with GITR-Fc did not impact the expression density of GITR on ILC1s (Figure 6A). The analysis of the ILC1 functionality revealed that triggering GITR as a result of DTA-1 treatment did not significantly impact the expression densities of IFN-γ and TNF-α. However, DTA-1 treatment resulted in significantly increased frequencies of IFN-γ^+^ and TNF-α^+^ ILC1s as compared to isotype-treated infected animals (Figure 6B). Next to the changes in the cytokine expression profile, the analysis of the surface activation marker TRAIL revealed marginal reduction in both, the expression density and frequency. GITR-Fc treatment led to significantly increased expression of IFN-γ, but not TNF-α and TRAIL as compared to untreated-infected animals (Figure 6C). The analysis of IFN-γ^+^, TNF-α^+^ and TRAIL^+^ ILC1s revealed significantly increased frequencies for all three markers (Figure 6C). Thus, the obtained data suggest that during the course of influenza infection, the interaction of GITR-expressing ILC1s with GITR-L-expressing cells shapes ILC1 functionality.

### Cross-Talk Between IAV PR8 H1N1-Infected DCs, GITR^lo^ ILC1s, and CD8 T Cells

**Figure 7:**
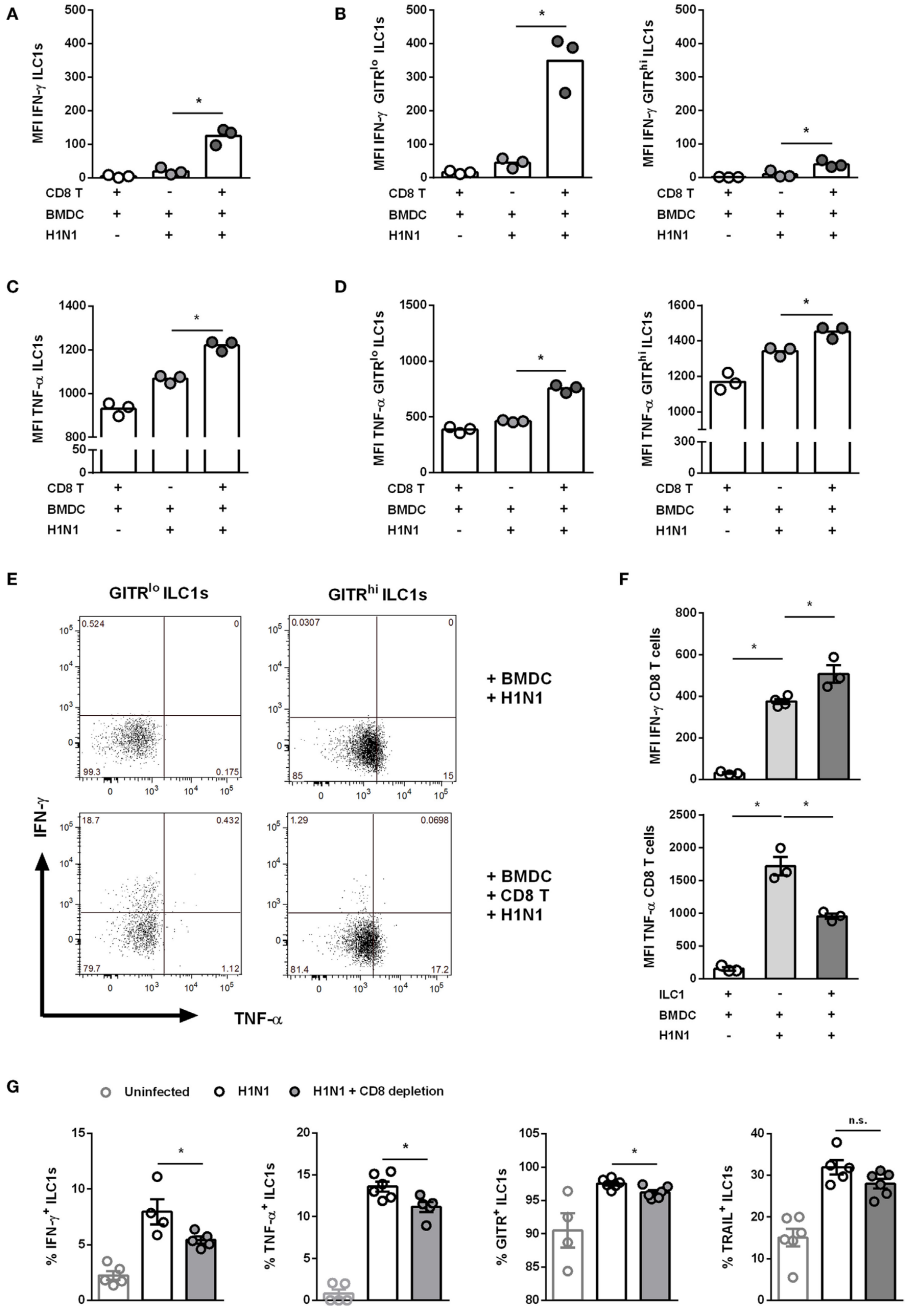
Cross-talk of ILC1s with BMDCs and CD8 T cells during H1N1 infection. FLT-3 ligand-differentiated BMDCs were infected with the SIINFEKL expressing H1N1 PR8 influenza strain at a MOI of 1. *In vitro*-generated ILC1s were cultured overnight with infected or uninfected BMDCs and CD8 T cells derived from OT I mice at a 1:1:1 ratio. Surface markers and cytokines were stained for flow cytometry analysis after 3 h of incubation in media with brefeldin and monensin. MFI of **(A,B)** IFN-γ and **(C,D)** TNF-α expression by ILC1s depending on the level of GITR expression. **(E)** Representative two-dimensional FACS plots of IFN-γ vs. TNF-α from GITR^lo^ and GITR^hi^ ILC1s in the absence or presence of CD8 T cells. **(F)** MFI of IFN-γ and TNF-α-expressing CD8 T cells. CD8 T cells were depleted before and 3 days after H1N1 PR8 infection (2 × 10^3^ ffu/animal) of wild-type mice by i.p. administration of CD8 T cell-depleting antibodies (200 μg/animal). Lung-derived lymphocytes of infected wild-type mice were analyzed by flow cytometry 6 dpi with regard to markers related to ILC1 activation and functionality. **(G)** Frequencies of IFN-γ^+^, TNF-α^+^, TRAIL^+^, and GITR^+^ lung-derived ILC1s. Bars with scatter plots represent the mean ± SEM and the *in vitro* (*n* = 3–4 technical replicates) and *in vivo* (*n* = 5–6) data are representative from one out of two independent experiments. Asterisks denote significant values as calculated by nonparametric Kruskal–Wallis test (Dunn’s posttest) (*in vitro* data) or One-way ANOVA (*in vivo* data); ****p ≤ 0.0001; ***p ≤ 0.001; **p ≤ 0.01; *p ≤ 0.05; n.s., not significant.

In addition to their interaction with DCs, ILC1s might also cross-talk with adaptive immune cells, thereby directing antiviral responses. CD8 T cells are crucial in clearing influenza infection in the absence of antibody responses (29). Thus, the H1N1 PR8 *in vitro* infection model was exploited to assess whether ILC1s cross-talk with CD8 T cells (Gating strategy; Figure S9A in Supplementary Material). To this end, the H1N1 PR8 strain expressing the CD8 ovalbumin peptide SIINFEKL was used for *in vitro* infection of BMDCs, which were subsequently cocultured with *in vitro*-generated ILC1s and OT I-derived CD8 T cells. The coculture of ILC1s with CD8 T cells and H1N1-infected BMDCs revealed that IFN-γ and TNF-α secretion by ILC1s was significantly enhanced as compared to cocultures of infected BMDCs and ILC1s without CD8 T cells (Figures 7A,C). Furthermore, GITR^lo^ ILC1s were found to be the main IFN-γ producers as compared to GITR^hi^ ILC1s (Figures 7B,E). Significantly increased levels of TNF-α were detected in both GITR^lo^ and GITR^hi^ ILC1s. However, the increment in TNF-α secretion upon coculture with infected BMDCs and CD8 T cells as compared to infected BMDCs alone was slightly higher in GITR^lo^ ILC1s (fold change: 1.63) than in GITR^hi^ ILC1s (fold change: 1.097) (Figures 7D,E). It needs to be considered that GITR^hi^ ILC1s already exhibited a high basal level of TNF-α secretion, even in absence of the cross-talk partners. Coculture of infected BMDCs with both CD8 T cells and ILC1s resulted in significantly enhanced BMDC maturation as compared to cocultures with CD8 T cells or ILC1s alone, as shown by the increased expression of CD80, CD86 and MHC cl. II (Figure S9C in Supplementary Material).

The coculture of CD8 T cells and ILC1s with infected BMDCs revealed that in this experimental model ILC1s also provide help to CD8 T cells. An enhanced IFN-γ secretion by CD8 T cells was observed as compared to cocultures of infected BMDCs together with CD8 T cells alone (Figure 7F). In contrast, the secretion of TNF-α by CD8 T cells was reduced upon coculture with ILC1s and infected BMDCs as compared to the coculture without ILC1s. These data suggest that H1N1-induced cross-talks between BMDCs, GITR^lo^-expressing ILC1s, and CD8 T cells imprint the cytokine profile of innate and adaptive immune cells. To assess the impact of ILC1 cross-talk with CD8 T cells in the course of H1N1 infection *in vivo*, ILC1s and CD8 T cells were co-transferred into RAG2^−/−^γc^−/−^ mice that were subsequently infected with the H1N1 PR8 strain expressing the CD8 ovalbumin peptide SIINFEKL. H1N1 infection of RAG2^−/−^γc^−/−^ mice adoptively transferred with CD8 T cells alone or together with ILC1s revealed that ILC1s did not additionally contribute to reduced viral loads in the lungs (Figure S10A in Supplementary Material). CD8 T cell-supplemented RAG2^−/−^γc^−/−^ mice showed reduced lung viral loads 3 dpi as compared to mice without CD8 T cells. To further elucidate the observed findings, an additional *in vivo* infection approach was used to study the impact of CD8 T cells on ILC1 functionality. For this, a CD8 T cell-depleting antibody was administered i.p. followed by i.n. H1N1 infection of wild-type mice. The depletion efficacy was confirmed by flow cytometry analysis of blood samples (Figure S10C in Supplementary Material). Influenza infection of CD8 T cell-depleted mice resulted in a reduction of body weight similar to the infected non-depleted mice (weight curve; Figure S10D in Supplementary Material). Lung-derived ILC1s were subsequently analyzed regarding their functionality. At day 3 post infection, the frequencies of IFN-γ^+^, TNF-α^+^, TRAIL^+^, and GITR^+^ ILC1s were increased upon H1N1 infection, however, no significant differences were observed upon CD8 T cell depletion (Figure S10E in Supplementary Material). Interestingly, 6 dpi, lung-derived ILC1s displayed altered functionality due to the absence of CD8 T cells. The frequencies of IFN-γ^+^ and TNF-α^+^ ILC1s significantly decreased upon CD8 T cell depletion as compared to infected but CD8 T cell-sufficient mice (Figure 7G). Next to the altered cytokine profile, decreased frequencies of GITR^+^ ILC1s were observed upon CD8 T cell depletion as compared to infected CD8 T cell-competent mice. No significant impact of CD8 T cell depletion on the frequency of TRAIL^+^ ILC1s was detected (Figure 7G). CD8 T cell depletion showed an effect on the frequencies of functional ILC1s, but not on the absolute numbers (data not shown). The observed results suggest that CD8 T cells communicate with ILC1s and thereby contribute to the functional activation of lung ILC1s during influenza infection.

### Cross-Talk Between H1N1-Infected DCs, GITR^lo^ ILC1s, and CD4 T Cells

**Figure 8:**
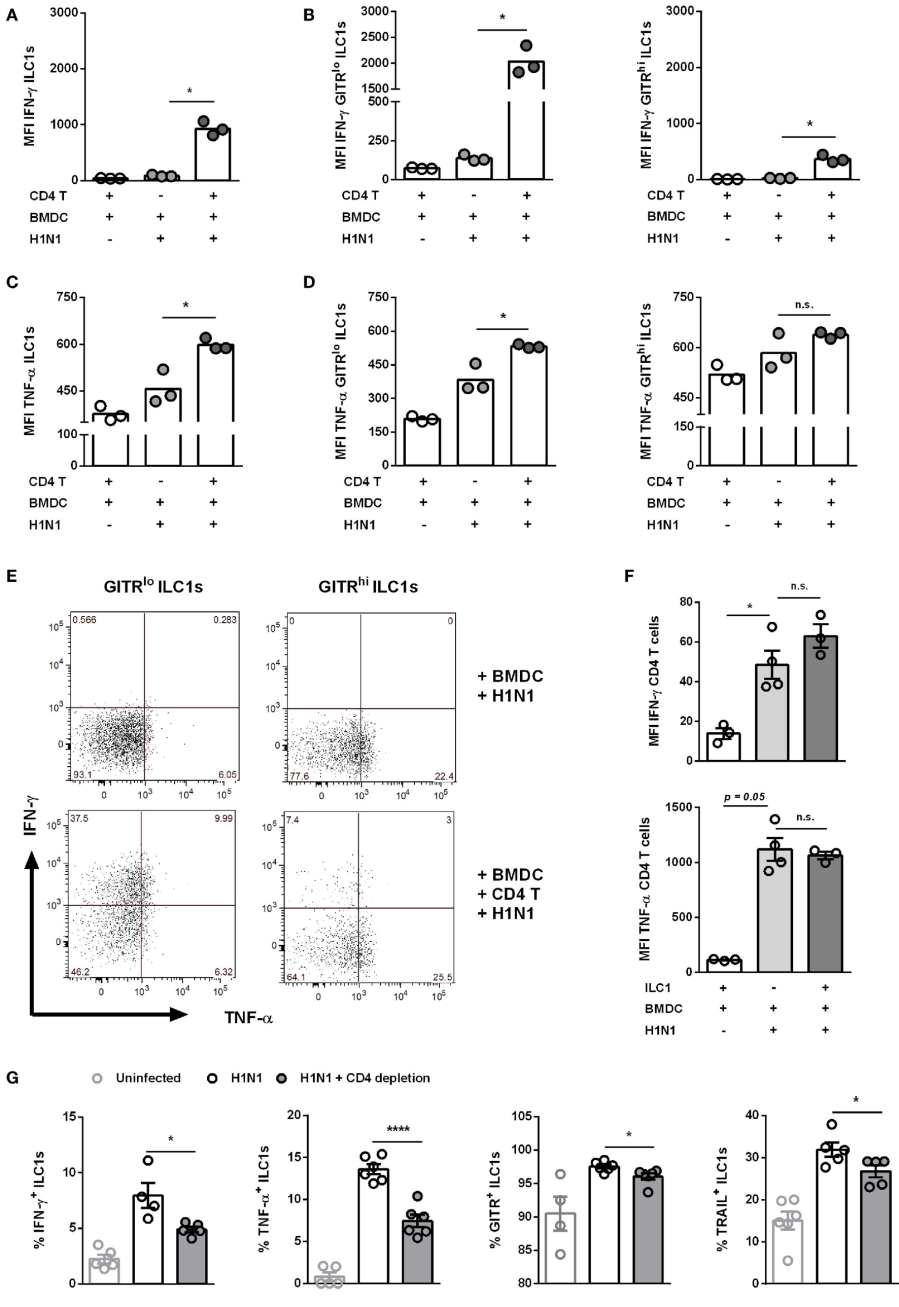
CD4 T cells contribute to enhanced ILC1 functionality during H1N1 infection *in vitro* and *in vivo*. FLT-3 ligand-differentiated BMDCs were infected with the OVA peptide (aa323–393)-expressing H1N1 PR8 influenza strain at a MOI of 1. *In vitro*-generated ILC1s were cocultured overnight with infected or uninfected BMDCs and CD4 T cells derived from OT II mice at a 1:1:1 ratio. Staining of surface makers and cytokines for flow cytometry analysis after 3 h of incubation in media with brefeldin and monensin. MFI of **(A,B)** IFN-γ and **(C,D)** TNF-α expression by ILC1s depending on the level of GITR expression. **(E)** Representative two-dimensional FACS plots of IFN-γ vs. TNF-α from GITR^lo^ and GITR^hi^ ILC1s in the absence or presence of CD4 T cells. **(F)** MFI of IFN-γ and TNF-α-expressing CD4 T cells. CD4 T cells were depleted before and 3 days after H1N1 PR8 infection (2 × 10^3^ ffu/animal) of wild-type mice by i.p. administration of CD4 T cell-depleting antibodies (200 µg/animal). Lung-derived lymphocytes of infected wild-type mice were stained for flow cytometry analysis 6 dpi with regard to markers related to ILC1 activation and functionality. **(G)** Frequencies of IFN-γ^+^, TNF-α^+^, TRAIL^+^, and GITR^+^ lung-derived ILC1s. Bars with scatter plots represent the mean ± SEM and the *in vitro* (*n* = 3–4 technical replicates) and *in vivo* (*n* = 5–6) data are representative from one out of two independent experiments. Asterisks denote significant values as calculated by nonparametric Kruskal–Wallis test (Dunn’s posttest) (*in vitro* data) or One-way ANOVA (*in vivo* data); ****p ≤ 0.0001; ***p ≤ 0.001; **p ≤ 0.01; *p ≤ 0.05; n.s., not significant.

Next to CD8 T cells, CD4 T cells play a broad role in the course of IAV infection by differentiating into diverse effector T helper subsets as well as promoting the generation of neutralizing antibodies, among other critical clearance mechanisms (29). To evaluate the potential cross-talk between ILC1s and CD4 T cells during IAV infection *in vitro*, the H1N1 PR8 strain expressing the ovalbumin peptide (aa323–aa339) specific for CD4 T cells was utilized for BMDC infection (Gating strategy; Figure S9B in Supplementary Material). Similar to the above reported findings concerning CD8 T cells, the addition of CD4 T cells to the H1N1 PR8 infection culture system boosted IFN-γ and TNF-α secretion by ILC1s (Figures 8A,C). GITR^lo^ ILC1s represented the more functional subset of ILC1s as compared to GITR^hi^ ILC1s with regard to IFN-γ (Figures 8B,E). Significantly increased levels of TNF-α were secreted by both GITR^lo^ and GITR^hi^ ILC1s. The increment in TNF-α secretion upon coculture with infected BMDCs and CD4 T cells as compared to infected BMDCs alone was marginally higher in GITR^lo^ ILC1s (fold change: 1.38) than GITR^hi^ ILC1s (fold change: 1.09) (Figures 8D,E). However, as observed for the cocultures with CD8 T cells, GITR^hi^ ILC1s exhibited already a high basal level of TNF-α secretion. Infected BMDCs cocultured with CD4 T cells and ILC1s displayed enhanced expression of the maturation markers CD80, CD86 and MHC cl. II as compared to infected BMDCs cocultured with CD4 T cells in the absence of ILC1s (Figure S9D in Supplementary Material). The analysis of the functional profile of CD4 T cells revealed that ILC1s are dispensable for the induction of IFN-γ and TNF-α in this experimental *in vitro* infection model (Figure 8F). To assess the impact of ILC1 cross-talk with CD4 T cells in the course of H1N1 infection *in vivo*, ILC1s and CD4 T cells were cotransferred into RAG2^−/−^γc^−/−^ mice that were subsequently infected with a H1N1 strain expressing the CD4 ovalbumin peptide aa232–339. The adoptive transfer studies revealed that CD4 T cells alone were not sufficient to control viral infection, whereas the addition of ILC1s resulted in a significantly decreased viral burden (Figure S10B in Supplementary Material). However, ILC1s transferred without CD4 T cells resulted in an even more pronounced reduction of the lung viral load. Thus, ILC1s seem to contribute to the control of viral infection as demonstrated by reduced viral loads following H1N1 infection (Figure S10B in Supplementary Material). To further strengthen our observation, an additional *in vivo* H1N1 infection model was applied to study the impact of CD4 T cells on ILC1 functionality. To this end, a CD4 T cell-depleting antibody was administered i.p. followed by i.n. influenza infection. The depletion efficacy was confirmed by flow cytometry analysis of blood samples (Figure S10C in Supplementary Material). Influenza infection of CD4 T cell-depleted mice resulted in a reduction of body weight similar to infected non-depleted mice (weight curve; Figure S10D in Supplementary Material). Lung-derived ILC1s were subsequently analyzed regarding their functionality. At day 3 post infection, CD4 T cell depletion did not significantly impact the frequency of functional ILC1s. A minor increase was observed in the absolute numbers of IFN-γ^+^, TNF-α^+^, TRAIL^+^, and GITR^+^ ILC1s in the absence of CD4 T cells as compared to infected but CD4 T cell-sufficient animals (data not shown). At day 6 post infection, depletion of CD4 T cells resulted in significantly reduced frequencies of IFN-γ^+^ and TNF-α^+^ ILC1s (Figure 8G). The analysis of surface activation markers revealed that the depletion of CD4 T cells also resulted in decreased frequencies of TRAIL^+^ and GITR^+^ ILC1s (Figure 8G). In addition, H1N1 infection induced significantly elevated absolute counts of IFN-γ^+^, TNF-α^+^, GITR^+^, and TRAIL^+^ ILC1s 6 dpi. Depletion of CD4 T cells led to significantly reduced numbers of these activated ILC1s (data not shown). Therefore, the obtained data suggest that CD4 T cells engage in a complex interaction with GITR^+^ ILC1s and regulate ILC1 functionality in the combat against influenza infection.

## Discussion

Respiratory infections caused by the influenza virus represent a major public health concern, leading to a significant death toll and economic burden worldwide. Due to antigenic drift and antigenic shift, current seasonal influenza vaccines require annual adaptation and reformulation. Furthermore, current influenza vaccines are not able to induce long lasting immunity and they are poorly immunogenic in some population groups at high risk for severe infections (e.g. elderly). Thus, there is an urgent need to develop novel or improved vaccines against influenza. Innate immunity marks the first line of defense against infections and in this context ILC1s are emerging effectors. It was demonstrated that HIV-infected individuals display decreased ILC1s numbers in blood at early time points after infection, thereby suggesting a role of ILC1s in initial viral clearance (30). However, the impact of ILC1s for acute viral infections, such as influenza, remains relatively unknown. In the present study, the role of ILC1s in the course of IAV infection and the identification of potential interaction partners of both innate and adaptive immunity were addressed.

Influenza infection of wild-type female mice resulted in activation and increased functionality of lung ILC1s as demonstrated by their enhanced production of IFN-γ and TNF-α early after infection. Here, it needs to be considered, that gender specific differences might result in different susceptibilities to influenza infection (31). Previous reports showed that ILC1s induce IFN-γ- and TNF-α-mediated protection against parasitic as well as bacterial infections (6, 7). In the course of the IAV infection, the relevance of innate IFN-γ secretion has been demonstrated by several groups. In this context, NK cell-derived early IFN-γ was shown to be crucial for DC migration as well as optimal activation of CD8 T cell responses (8, 32). TNF-α was indicated to be beneficial early during influenza infection either by a direct antiviral effect on lung-epithelial cells or by activating macrophages and inducing DC maturation (33). Thus, ILC1-derived IFN-γ and TNF-α might contribute to early viral control by either direct antiviral effects or the activation of other bystander immune cells. The observation of early IFN-γ and TNF-α secretion by ILC1s 3 dpi suggests that proinflammatory cytokines produced by the ILC1s play a major role in initiating antiviral defense responses. This hypothesis is further supported by the finding of reduced viral titers 3 dpi in ILC1-sufficient mice as compared to mice lacking ILC1s. A recent published study could not detect differences in the viral load following the depletion of ILC1s including NK cells using an α-NK1.1 antibody (12). The contrary findings might be due to the use of a different Rag1^−/−^ mouse strain and that the viral load was determined at later time points (10 dpi). In addition to IFN-γ and TNF-α, local elevations of the type 1 cytokines IL-12 and IL-18 were detected in the BAL of infected mice. This is consistent with reports demonstrating that influenza-induced adaptive immunity is shaped by innate type 1 responses (26, 27). Recently, ILC1s were shown to become activated *in vitro* by IL-12 and IL-18 (34). Thus, the present findings provide indirect evidence for IAV-induced ILC1 activation.

ILC1s were shown to express the death receptor TRAIL and integrin CD49a at steady state (35). In the present study, IAV induced increased frequencies of TRAIL^+^ and CD49a^+^ lung ILC1s, as well as expression densities of TRAIL and CD49a. The observed enhanced expressions of TRAIL and CD49a by adoptively transferred ILC1s provides further evidence for influenza-induced ILC1 activation. TRAIL expressed on NK cells was shown to be up-regulated post influenza infection resulting in antiviral cytotoxicity, thereby contributing to protection (36). TRAIL^+^ NK cells were also demonstrated to kill DCs thereby functionally regulating DC responses (37). Furthermore, CD8 T cells expressing TRAIL were demonstrated to kill influenza-infected cells, thereby representing an important effector mechanism of protective immunity (38). Hence, the observed enhanced expression of TRAIL and increased frequencies of TRAIL^+^ lung ILC1s at early time points post infection might contribute toward protection against IAV infection *via* direct killing of infected cells or by modulating DC responses. CD49a expressed by T cells was demonstrated to promote T cell retention and survival in extra-lymphoid tissues during influenza infection (39, 40). Thus, CD49a expressed by ILC1s might also support ILC1 maintenance within the inflamed lung tissue.

The plasticity of ILCs renders it difficult to define the origin of specific subpopulations. Thus, although the ILC1s characterized here *in vivo* are based on the commonly accepted definition of being NKp46^+^T-bet^+^Eomes^-^RORγt^-^, they might also arise from ILC2s and/or ILC3s due to the cytokine milieu induced during IAV infection (41). Recently, an influenza-induced differentiation of ILC2s into an ILC1-like phenotype 7 dpi was reported. IFN-γ secreted from these ILC2-derived ILC1s contributed toward influenza-induced inflammation (34). However, this trans-differentiation was gradual and it was not evident from the study whether ILC2s can transdifferentiate into ILC1s at early time points after influenza infection. Thus, the observation of an enhanced cytokine profile and activation status already 2 dpi strongly argues in favor of an original ILC1 rather than an ILC2-derived ILC1 subpopulation. Likewise, an IL-12- and IL-18-induced trans-differentiation of ILC3s into ILC1s was described (42). However, to date plasticity of ILC3s is reported for lamina propria but not lung ILC3s. To explicitly ensure the identification of the origin of ILC1s, fate mapping studies are required. Influenza infection-induced IL-12 and IL-18 might result in the activation of *de novo* ILC1s and/or transdifferentiation of ILC2s and/or ILC3s into IFN-γ-producing ILC1s. In this way, an early innate ILC1 response to influenza might contribute to the antiviral defense. Furthermore, it is reported that IFN-γ restricts ILC2-mediated pathology (43). Thus, ILC1-derived IFN-γ might counteract influenza-induced immune pathology.

Innate immune responses are a pre-requisite for the generation of protective adaptive immunity. DCs serve as potent APCs and display a central role in the induction of specific adaptive immune responses against the influenza virus (44). Using the H1N1 *in vitro* infection system, a potential bidirectional cross-talk of ILC1s and DCs was revealed. It is known that DCs can potentiate NK cell functionality *via* enhanced IL-12 and IL-15 production after influenza infection (45). Furthermore, DC-derived IL-12 and IL-18 can also induce the cytotoxic and effector functions of NK cells (26, 27). However, a communication between DCs and ILC1s during IAV infection was not reported so far. The present study demonstrates that ILC1s can contribute to DC maturation during IAV infection. The observed decrease of CD40 might be due to internalization with its ligand post interaction as described for other immune cells (46). Interestingly, DCs seem to potentiate ILC1 functionality as shown by enhanced IFN-γ and TNF-α secretion. These results assume that influenza-activated DCs might boost type 1 responses of ILC1s and reciprocally activated ILC1s can potentiate DC maturation.

Similar to NK cells, the cross-talk of ILC1s with DCs might impact the adaptive immune system. The observation of marginally increased lung viral loads 6 dpi in ILC1-sufficient RAG2^−/−^γc^−/−^ mice might be ascribed to the absence of adaptive immune cells. Adoptive transfer studies of CD8 T cells and ILC1s followed by influenza infection revealed that ILC1s do not directly contribute to CD8 T cell-mediated viral clearance. The adoptive transfer of CD4 T cells with ILC1s resulted in marginally reduced viral titers. Interestingly, reduction in viral titers in mice adoptively transferred with ILC1s alone was much higher as compared to mice which received both CD4 T cells and ILC1s. This observation suggests that CD4 T cells can regulate ILC1 functionality in the course of influenza infection. In this context, regulatory T cells might play a major role and need to be considered for further studies. Furthermore, due to the lack of several key innate and adaptive immune players, an inherent feature of the used mouse strain (RAG2^−/−^γc^−/−^), the immune response of the remaining immune cell populations (APCs) might be significantly altered. Nevertheless, the performed *in vitro* infection studies suggest an extensive cross-talk between infected BMDCs, ILC1s, and different T cell subpopulations. ILC1s seem to provide help to CD8 T cells by enhancing their IFN-γ production, whereas they induced a reduction in TNF-α expression. This reduction hints toward a regulatory role of ILC1s thus preventing immune pathology as a result of excessive cytokine secretion. These findings on ILC1-mediated regulation are supported by a study which demonstrated that CD49a^+^ ILC1s regulate the priming of antiviral CD8 T cells in the course of liver infection by inhibiting the influx of NK cells (47). Interestingly, ILC1s displayed an enhanced cytokine production, assessed by IFN-γ and TNF-α, due to the presence of CD8 or CD4 T cells in the H1N1-DC-coculture model. These findings were further confirmed by *in vivo* depletion experiments which revealed that the depletion of CD4/CD8 T cells resulted in decreased ILC1 functionality. Interestingly, these findings were only observed 6 dpi but not at earlier time points suggesting a cross-talk of ILC1s with activated T cells. However, it needs to be considered that CD4/CD8 T cell depletion might influence other immune cell populations. Therefore, it remains elusive whether the ILC1–T cell cross-talk is due to a direct or indirect effect. Furthermore, an increased IFN-γ secretion by ILC1s could result in enhanced CD8 T cytotoxicity against infected cells (32). It was reported that CD8 T effector cells unable to produce IFN-γ lead to severe lung damage as compared to IFN-γ-producing CD8 T cells (48). Therefore, the observed effect of elevated IFN-γ secretion by CD8 T cells due to the reciprocal cross-talk with ILC1s might hamper immune-mediated lung damage. Increased IFN-γ secretion by CD8 T cells might also lead to the infiltration of other immune cells, such as CD4 T cells. These CD4 T cells could provide help to promote antibody-mediated protection (29). Considering that IAV-infected BMDCs displayed higher maturation in coculture with ILC1s and either CD8 or CD4 T cells, ILC1s might communicate with CD4/CD8 T cells in a DC-dependent manner during influenza infection.

Although immune cell activation is a prerequisite to combat viral infections, excessive stimulation can result in undesired side effects like immune pathology. Here, a potential novel mechanism involved in the regulation of ILC1 functionality was investigated. In the present study, increased GITR expression on lung-derived ILC1s following H1N1 infection *in vivo* was detected. Furthermore, GITR-expressing ILC1s (GITR^+^) were functionally more active as compared to ILC1s lacking GITR (GITR^-^) expression. DCs isolated from dLNs were found to display elevated levels of the GITR-L, which hints toward a potential interaction between GITR^+^ ILC1s and GITR-L^+^ DCs. A previous study mapped the kinetics of GITR and GITR-L up-regulation on CD4 T cells and APCs, respectively, after herpes simplex virus infection and emphasized the role played by the GITR-GITR-L signaling in the regulation of virus-induced immune pathology (49). However, GITR was also demonstrated to serve as negative regulator of NK cells by suppressing proinflammatory cytokine secretion and boosting NK cell apoptosis (50). Thus, an ambivalent dual role for GITR in the context of immune regulation should be considered. The role of GITR signaling has been extensively studied on T cells, whereas non-T cells are less well characterized. For CD4 and CD8 T cells, intrinsic downstream signaling was shown to be crucial for the expansion, survival and cytokine production of virus specific T cells (51, 52). Our data suggest a correlation between increased ILC1 functionality and enhanced GITR expression in the course of IAV infection. Furthermore, the GITR–GITR-L interaction might contribute to ILC1 survival and influenza-mediated cytokine secretion. The presented results on GITR–GITR-L signaling using the DTA-1 antibody during influenza infection suggest that the stimulation of the GITR-intrinsic signaling not only results in reduced lung viral burden but also leads to increased ILC1 functionality. Furthermore, the observation that GITR-Fc treatment also results in increased cytokine production by ILC1s hints that the GITR-GITR-L interaction might contribute toward anti-influenza immunity. Triggering the GITR-L with the GITR-Fc fusion protein also results in a higher frequency of TRAIL^+^ ILC1s suggesting that the GITR signaling supports TRAIL-induced cytotoxicity. It needs to be pointed that additional stimulation through the use of external agonists/fusion protein could result in further enhanced functional ILC1 responses. Further, an impact of DTA-1 or GITR-Fc on bystander immune cells cannot be excluded. To completely ascertain the role of the GITR-GITR-L signaling in the course of an influenza infection, experiments using GITR^−/−^ mice might be beneficial. However, GITR is crucial in maintaining immune regulation and the phenotype of GITR knock out mice might impact other immune functions (53).

Influenza-activated GITR^+^ ILC1s displayed differential GITR expression and could be differentiated into GITR^hi^ and GITR^lo^ ILC1s. Interestingly, GITR^hi^ ILC1s represented a more exhausted activation profile as compared to GITR^lo^ ILC1s. The differential GITR expression on ILC1s *in vivo* was observed 2 and 3 dpi, whereas most ILC1s investigated 6 dpi were GITR^lo^. Similarly, *in vitro* generated ILC1s displayed two different GITR expression profiles. IL-15 was previously shown to result in overexpression of GITR on bone marrow memory CD8 T cells, thereby promoting their survival (54). Therefore, IL-15 added to the cultures could drive differential expression of GITR on *in vitro* generated ILC1s. Similar to the *in vivo* observation, major functional changes in ILC1 cytokine production were observed in GITR^lo^ ILC1s, but not GITR^hi^ ILC1s in the *in vitro* H1N1 PR8 infection model. This indicates that overexpression of GITR rendered ILC1s functionally less responsive toward stimulation by other immune cells. A gene expression study recently demonstrated GITR overexpression in acute and chronic HTLV-1-infected transformed CD4 T cells. However, the role of GITR expression was not explored (55). The present study also revealed a GITR-mediated ILC1 regulation upon IL-12 and IL-18 stimulation *in vitro*. The observed decrease of GITR expression upon addition of the GITR-Fc fusion protein to the H1N1 *in vitro* infection system might be caused by the binding of the fusion protein to the GITR-L, which is up-regulated on ILC1s after *in vitro* influenza infection. Previous studies showed that ligand engagement can modulate receptor expression (56). Our findings displayed that a partial reduction of GITR led to increased IFN-γ production. In addition, the reduced GITR expression on ILC1s resulted in significantly enhanced maturation of DCs. However, it cannot be ruled out that these effects could also be due to the binding of the Fc-blocking protein to the GITR-L on the DCs and their subsequent activation (25). It also needs to be pointed out that in this experimental setting complete GITR reduction was not achieved. Therefore, the findings might differ in the complete absence of GITR signaling. However, the obtained data identify GITR signaling as a novel mechanism involved in the regulation of the functional status of ILC1s during influenza infection.

In conclusion, the present study expands our understanding of the functional role played by ILC1s in viral infections. For the first time, an impact of lung ILC1s in the course of acute IAV infection was demonstrated. The observed contribution of ILC1s to viral clearance was most probably mediated by an enhanced activation status and elevated functionality. The findings of multireciprocal cross-talks between ILC1s and innate as well as adaptive immune cells during the course of IAV infection emphasize their up to now underestimated role. Interestingly, targeting the GITR signaling in regulatory T cells *via* agonistic antibodies is under clinical research for cancer immunotherapy (57). Murine studies currently address the effect of GITR targeting on CD8 T cells in chronic viral infections (57, 58). Thus, new approaches focusing on fine-tuning ILC1 functionality *via* differential GITR expression levels might represent a promising tool for the development of improved or new immune interventions against infections. However, further research is required to delineate the mechanisms of GITR-induced regulation of ILC1s and to dissect the communication of ILC1s with other immune cells. If ILC1s indeed imprint crucial aspects of adaptive antiviral immunity, they can be used as targets for prophylactic or therapeutic interventions against viral diseases.

## Ethics Statement

Mice were treated in consensus with local and European Community guidelines and were housed under specific pathogen-free conditions in individual ventilated cages with food and water *ad libitum*. The performed animal experiments were approved by the local government in Braunschweig, Germany (AZ: 33.42502-04-13/1281 and AZ: 33.19-42502-04-16/2280). All animal experimentation was performed under both institutional and national guidelines.

## Author Contributions

NV, PR, ST, TE, and BJC designed the experiments. NV performed experiments and acquired data. NV, PR, ST, and BJC analyzed data. TE provided reagents. NV and PR wrote the manuscript draft. CAG, ST, and PR discussed data and revised the final manuscript. All authors reviewed and edited the manuscript.

## Conflict of Interest Statement

The authors declare that the research was conducted in the absence of any commercial or financial relationships that could be construed as a potential conflict of interest.
