# Supplementary material for: Influenza-Activated ILC1s Contribute to Antiviral Immunity Partially Influenced by Differential GITR Expression
Source: Front Immunol. 2018 Mar 22;9:505. doi: 10.3389/fimmu.2018.00505 (PMC5874297; doi:10.3389/fimmu.2018.00505)
Supplement: Supplementary file 1 [file data_sheet_1.PDF]

Supplementary Material

Influenza-activated ILC1s contribute to anti-viral immunity partially influenced by differential GITR expression

Neha Vashist [1,2], Stephanie Trittel [1], Thomas Ebensen [1], Benedict J. Chambers [2] , Carlos A. Guzmán [1], Peggy Riese [1]

* Correspondence: Dr. Peggy Riese: Peggy.Riese@helmholtz-hzi.de

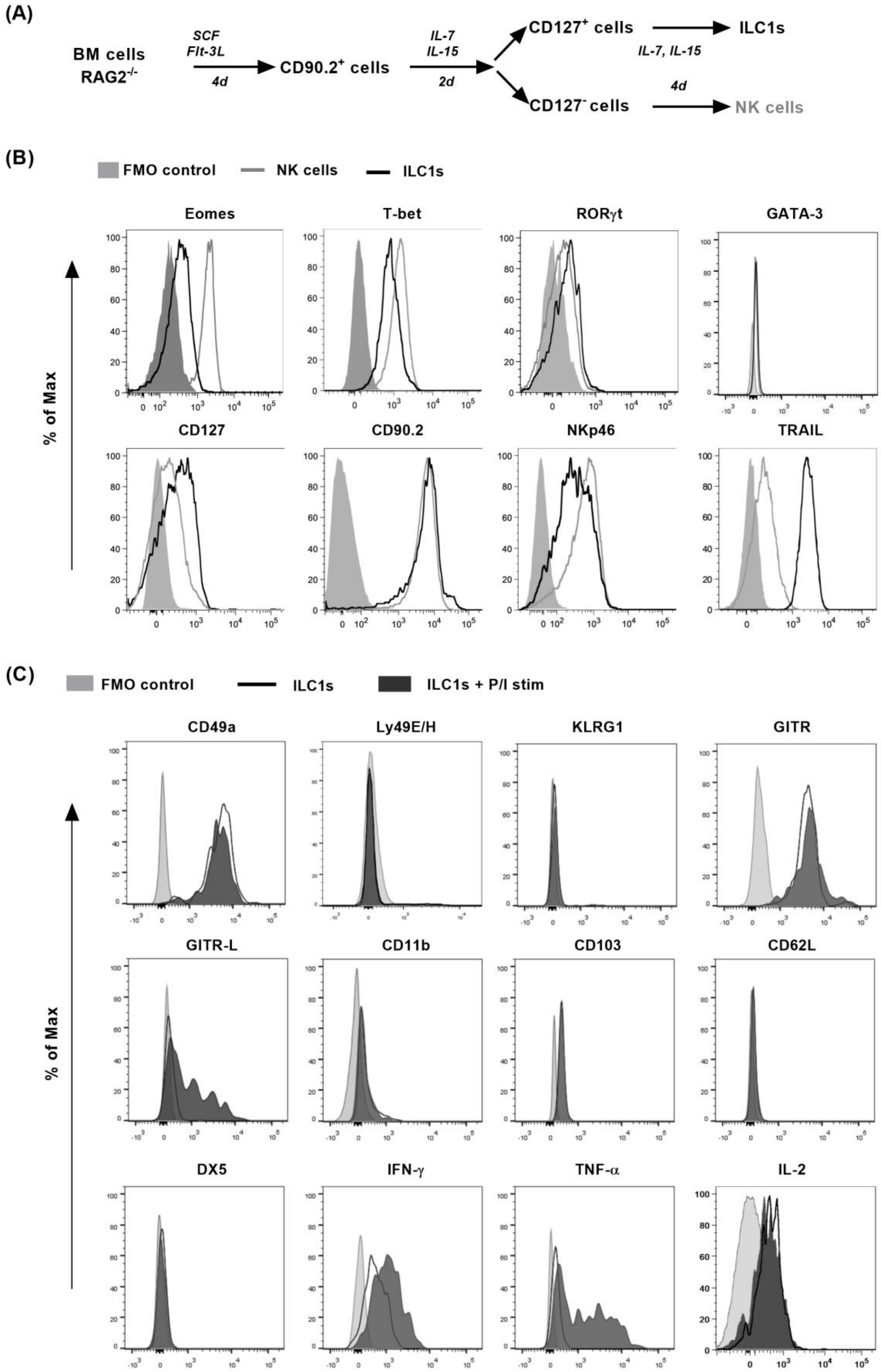

(A)
BM cells
RAG2-/-
SCF
Flt-3L
4d
CD90.2+ cells
IL-7
IL-15
2d
CD127+ cells
ILC1s
IL-7, IL-15
4d
CD127- cells
NK cells
(B)
FMO control
NK cells
ILC1s
Eomes
T-bet
RORγt
GATA-3
CD127
CD90.2
NKp46
TRAIL
% of Max
(C)
FMO control
ILC1s
ILC1s + P/I stim
CD49a
Ly49E/H
KLRG1
GITR
GITR-L
CD11b
CD103
CD62L
DX5
IFN-γ
TNF-α
IL-2
% of Max

Figure S1: Phenotypic characterization of *in vitro* generated ILC1s. Bone marrow cells were isolated from RAG2$^{-/-}$ and cultured with SCF & FLT-3 ligand (100ng/ml) for 4 days. Subsequently, CD90^{+} cells were magnetically enriched and cultured for 2 days with IL-7 and IL-15 (100ng/ml). Following, CD127^{+} as well as CD127^{-} cells were collected separately and again cultured with IL-7 and IL-15 for 4 days. (A) Flowchart describing ILC1 generation protocol and (B) representative histograms showing transcription factor staining (Eomes, T-bet, RORγt and GATA-3) comparing ILC1s and NK cells. Eomes^{-}T-bet^{+} ILC1sand Eomes^{+}T-bet^{+} NK cells were further analyzed for the surface expression of the identification markers CD127, CD90.2, NKp46, and the cytotoxicity marker TRAIL. (C) *In vitro*-generated ILC1s were stimulated for 1 h in media with PMA/Ionomycin and further for 3 h in medium containing brefeldin and monensin. Eomes^{-}T-bet^{+} ILC1s were analyzed for the surface expression of CD49a, Ly49s, KLRG1, GITR, GITR-L, CD11b, CD103, CD62L and DX5 as well as the expression of the cytokines IFN-γ, TNF-α and IL-2. The shown data are representative from one out of three independent experiments.

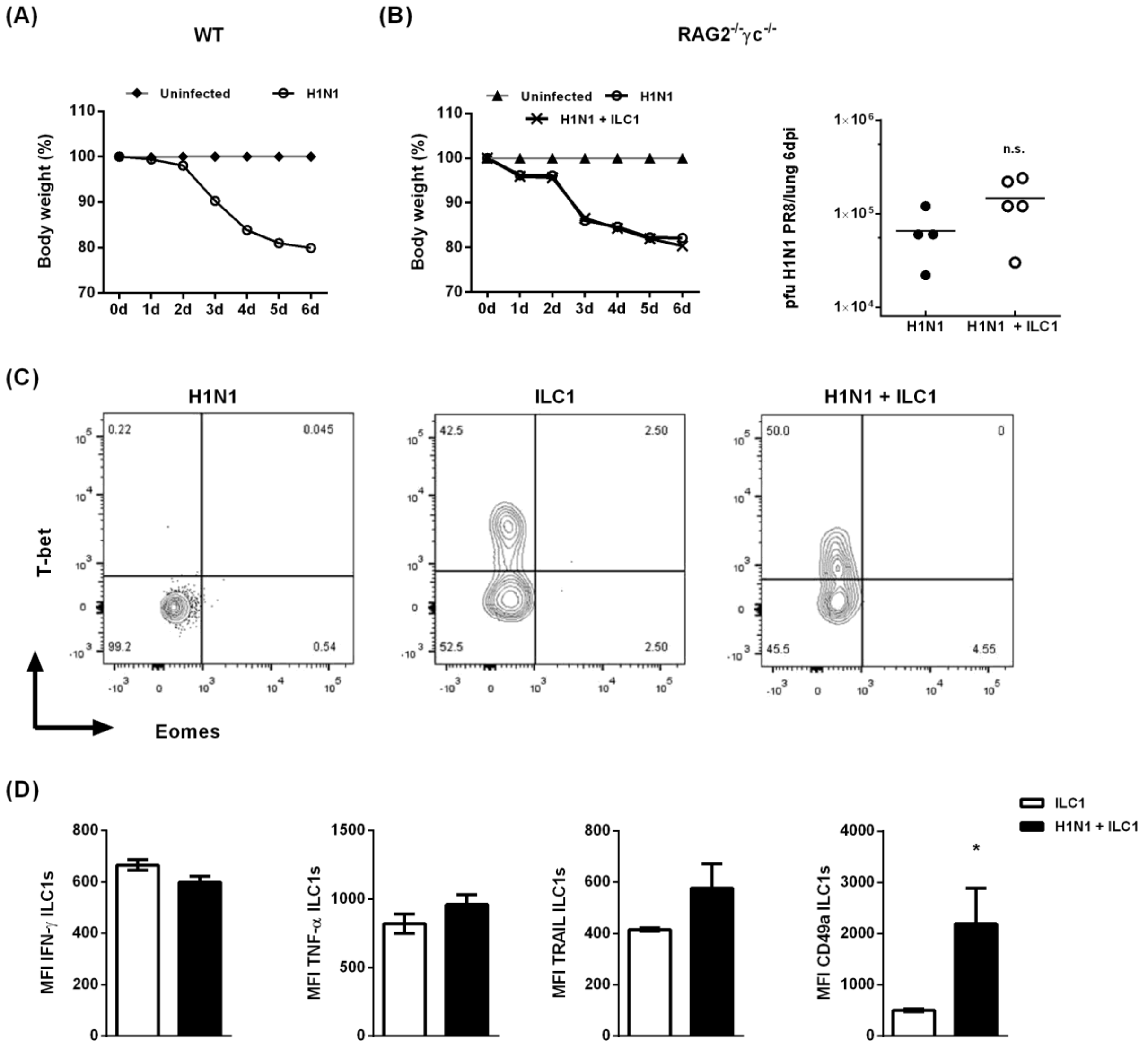

Figure S2: H1N1 infection results in ILC1 activation. Wild-type and RAG2$^{-/-}$γc$^{-/-}$ mice were infected i.n. with 2*10^{3} ffu of H1N1 PR8. (A) Body weight (%) displayed for wild-type mice. (B) Bodyweight (%) and viral loads for RAG2$^{-/-}$γc$^{-/-}$ mice post infection. Scatter plots represent viral loads of (*in vitro*-generated) ILC1-sufficient (2*10^{5} cells/animal) or -insufficient RAG2$^{-/-}$γc$^{-/-}$ mice 6 dpi (right panel; n= 4-5) as determined by foci assay and depicted as ffu per lung. Lymphocytes from ILC1-insufficient uninfected, ILC1-sufficient infected and uninfected lungs of RAG2$^{-/-}$γc mice were isolated 3 dpi. Lymphocytes from each group were pooled and stained for flow cytometry analysis with regard to markers related to ILC1 activation and functionality after 3 h of incubation in media with brefeldin and monensin. (C) Representative plots gated on CD45^{+}CD90^{+}CD127^{+}NKp46^{+} lung cells depicting T-bet^{+}Eomes^{-} ILC1s in infected ILC1-insufficient (H1N1), ILC1-sufficient uninfected (ILC1) and ILC1- sufficient infected (H1N1 +ILC1) RAG2$^{-/-}$γc$^{-/-}$ mice. (D) MFI of IFN-γ, TNF-α, TRAIL and CD49a expressed by

adoptively transferred *in vitro*-generated ILC1s derived from the lungs 3dpi. Asterisks denote significant values as calculated by unpaired, two-tailed Student's t-test as compared to uninfected samples; ****$p \leq 0.0001$; *** $p \leq 0.001$; ** $p \leq 0.01$; *$p \leq 0.05$; n.s., not significant.

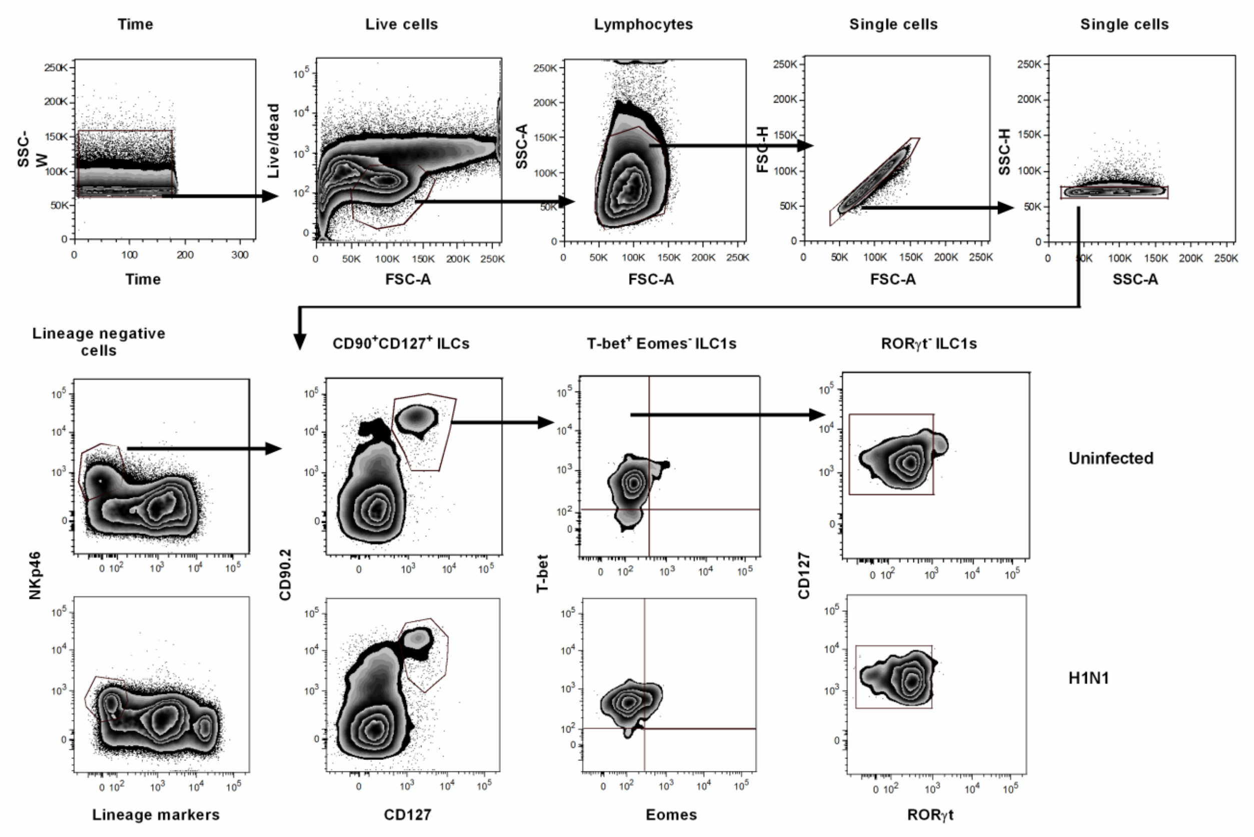

Figure S3: ILC1 gating strategy. Gating on SSC-W vs Time allowed the exclusion of measurement disturbances. Living cells were selected and lymphocytes were identified based on their forward and side scatter profile. Doublet cells and lineage positive cells (CD3, CD19, Gr1 and Ter-119) were excluded and *ex vivo* lung-derived ILC1s from uninfected and infected groups were identified as $NKp46^{+}CD90^{+}CD127^{+}T\text{-}bet^{+}Eomes^{-}ROR\gamma t^{-}$ cells. ILC1s were further used for analysis of phenotypic and functional markers.

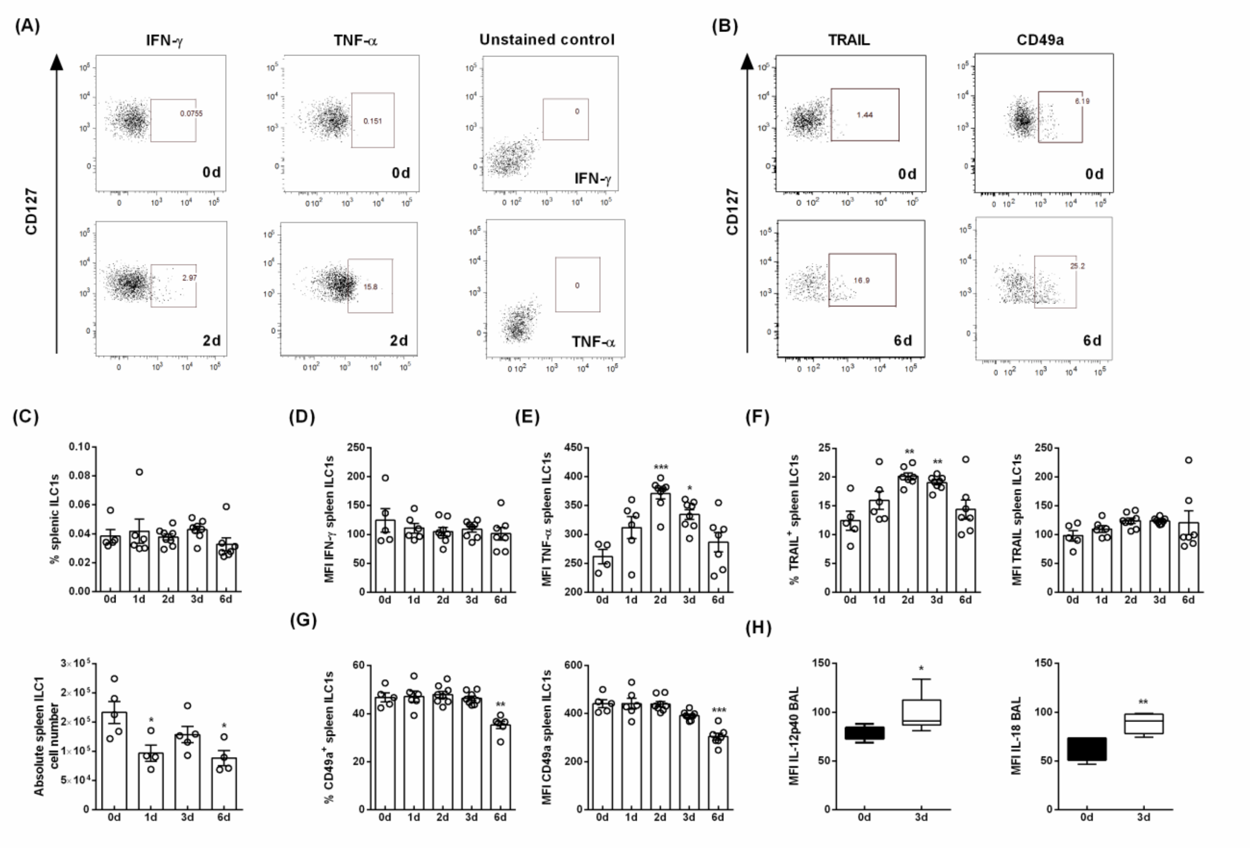

Figure S4: Lung and splenic ILC1 activation upon H1N1 PR8 infection. Wild-type mice were infected i.n. with $2*10^3$ ffu of H1N1 PR8. Lymphocytes derived *ex vivo* from lungs and spleens of infected wild-type mice were incubated at 37°C in medium containing brefeldin and monensin for 3 h before the flow cytometry staining was performed with regard to markers related to ILC1 activation and functionality at the indicated time points. (A) Representative plots of $CD127^+$ ILC1s derived from lungs of uninfected (0d) and infected (2d) groups showing IFN-γ and TNF-α secretion with unstained samples as controls. (B) Representative plots of $CD127^+$ ILC1s derived from lungs of control (0d) and infected (6d) groups showing TRAIL and CD49a expression. (C) Frequencies and absolute numbers of splenic ILC1s. MFI of (D) IFN-γ and (E) TNF-α expression and frequencies and MFI of (F) TRAIL and (G) CD49a expression by splenic ILC1s. Lavage buffer from the lungs (BAL) was collected for the determination of IL-12 and IL-18 by cytometric bead array (CBA). (H) MFI of IL-12 and IL-18 detected in BAL derived from uninfected and infected mice. Bars with scatter plots represent the MFI and range in frequency variation with the horizontal line drawn at the mean. Data are representative for one out of two independent experiments (n = 4-8). Asterisks denote significant values as calculated by unpaired, two-tailed Student's t-test (CBA) or One-way ANOVA (absolute counts, MFI and frequencies) as compared to uninfected samples; ****$p \leq 0.0001$; *** $p \leq 0.001$; ** $p \leq 0.01$; *$p \leq 0.05$.

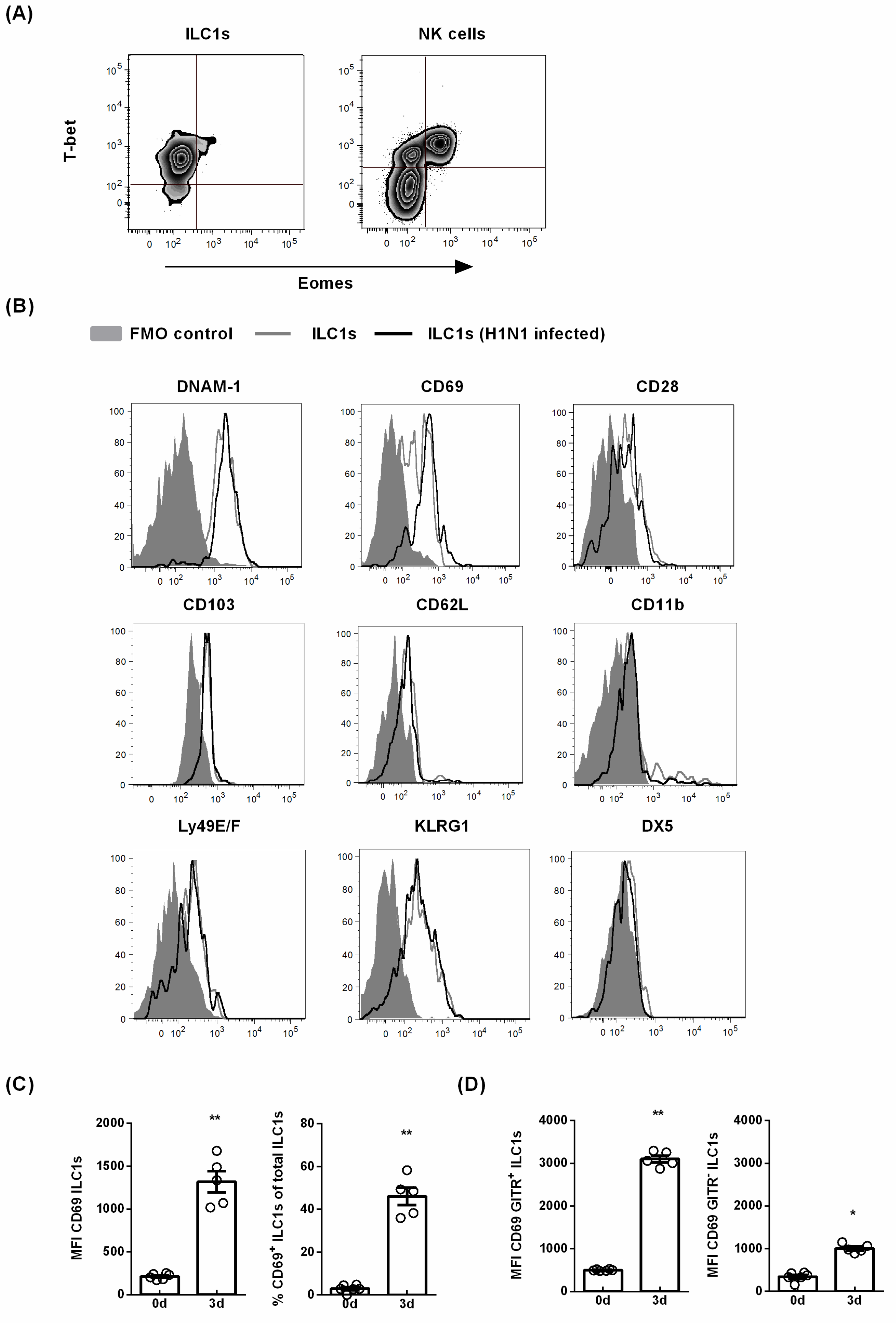
(A)
ILC1s
NK cells
T-bet
Eomes
(B)
FMO control
ILC1s
ILC1s (H1N1 infected)
DNAM-1
CD69
CD28
CD103
CD62L
CD11b
Ly49E/F
KLRG1
DX5
(C)
MFI CD69 ILC1s
% CD69+ ILC1s of total ILC1s
0d
3d
**
(D)
MFI CD69 GITR+ ILC1s
MFI CD69 GITR- ILC1s
*

Figure S5: Phenotypic characterization of *ex vivo*-derived lung ILC1s. Wild-type mice were infected i.n. with $2*10^3$ ffu of H1N1 PR8. Lymphocytes derived *ex vivo* from lungs of infected and uninfected wild-type mice were incubated at 37°C in medium containing brefeldin and monensin for 3 h before the flow cytometry staining was performed with regard to markers related to group 1 ILC identification, activation and functionality 3 dpi. (A) Representative FACS plots of ILC1s (left panel) and NK cells (right panel) comparing the expression of the transcription factors T-bet vs Eomes following gating on Lineage$^-$ (CD3,CD19, Gr1 and Ter-119) NKp46$^+$ cells derived from lungs of uninfected (0d) groups. (B) Representative histograms of CD127$^+$ ILC1s derived from lungs of control (0d) and infected (3d) groups showing DNAM-1, CD69, CD28, CD103, CD62L, CD11b, Ly49E/F, KLRG1 and DX5 expression. (C) MFI and frequencies of CD69$^+$ lung ILC1s derived from lungs of control (0d) and infected groups (3d). (D) MFI of CD69expression from GITR$^+$ and GITR$^-$ lung ILC1s. Bar with scatter plots represent the MFI and range infrequency variation with the horizontal line drawn at the mean. Data are representative for one out of two independent experiments (n = 4-6). Asterisks denote significant values as calculated by nonparametric Mann-Whitney's test as compared to uninfected samples; ****$p \leq 0.0001$; *** $p \leq 0.001$; ** $p \leq 0.01$; *$p \leq 0.05$.

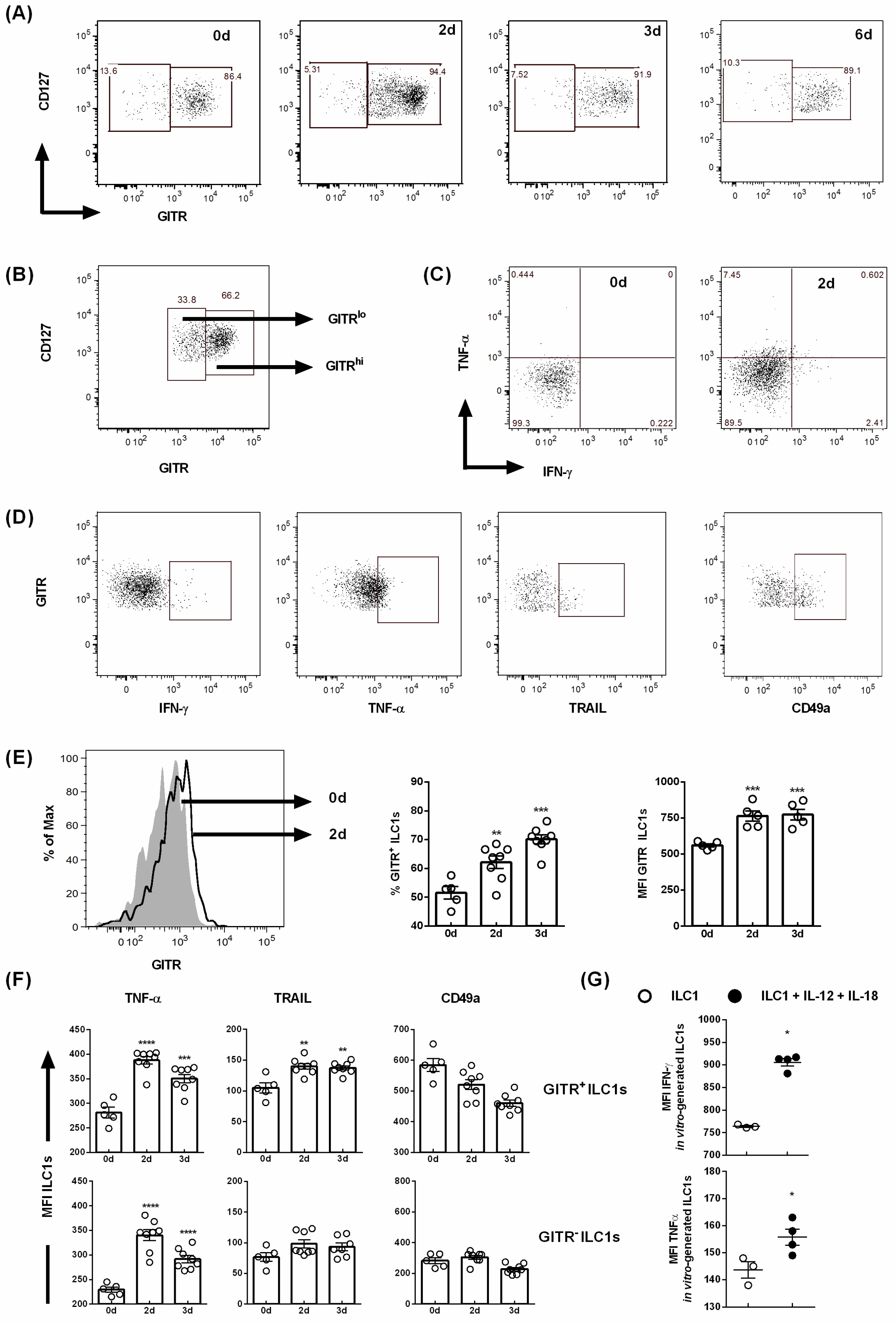

(A)
0d
2d
3d
6d
CD127
GITR
13.6
86.4
5.31
94.4
7.52
91.9
10.3
89.1
(B)
33.8
66.2
GITR^lo
GITR^hi
(C)
0.444
0
99.3
0.222
7.45
0.602
89.5
2.41
TNF-α
IFN-γ
(D)
IFN-γ
TNF-α
TRAIL
CD49a
(E)
% of Max
% GITR+ ILC1s
MFI GITR ILC1s
(F)
MFI ILC1s
GITR+ ILC1s
GITR- ILC1s
(G)
ILC1
ILC1 + IL-12 + IL-18
MFI IFN-γ in vitro-generated ILC1s
MFI TNFα in vitro-generated ILC1s

Figure S6: Increased GITR expression on lung and splenic ILC1s after *in vivo* H1N1 infection. Wild-type mice were infected i.n. with $2*10^3$ ffu of H1N1 PR8. Lung and splenic lymphocytes were incubated at 37°C in medium containing brefeldin and monensin for 3 h before the flow cytometry staining was performed with regard to markers related to ILC1 activation and functionality at the indicated time points. (A) Representative plots of $CD127^+$ ILC1s derived from lungs of control (0d) and infected groups (2d, 3d and 6d) depicting the flow cytometry gating for $GITR^+$ and $GITR^-$ ILC1s. (B) Representative plot of lung-derived $GITR^+$ ILC1s depicting the flow cytometry gating for $GITR^{lo}$ and $GITR^{hi}$ ILC1s. (C) Representative plot of $CD127^+$ ILC1s depicting the secretion of IFN-γ vs. TNF-α 0 and 2 dpi. (D) Representative plots of lung-derived $GITR^+$ ILC1s depicting the flow cytometry gating for IFN-γ^+, TNF-α^+, $TRAIL^+$ and $CD49a^+$ ILC1s. (E) Expression of GITR by splenic ILC1s (0d and 2d) depicted as representative histograms. Diagrams depict the frequencies and MFI of splenic GITR-expressing ILC1s (0d, 2d and 3d). (F) MFI of TNF-α, TRAIL and CD49a expressed by $GITR^+$ and $GITR^-$ splenic ILC1s. *In vitro*-generated ILC1s were stimulated with 100 ng/ml of IL-12 ± IL-18 for 48 h and subsequently incubated at 37°C in medium containing brefeldin and monensin for 3 h before the flow cytometry staining was performed with regard to markers related to ILC1 activation and functionality. (G) MFI of IFN-γ and TNF-α expression by *in vitro*-generated ILC1s post IL-12 ± IL-18 treatment. Bars with scatter plots represent the MFI and range in frequency variation with the horizontal line drawn at the mean. MFI data are representative from one out of two independent experiments (n = 5-8). Asterisks denote significant values as calculated by One-way ANOVA (*in vivo*) or nonparametric Mann-Whitney's test (*in vitro*) as compared to control samples; ****$p \leq 0.0001$; *** $p \leq 0.001$; ** $p \leq 0.01$; *$p \leq 0.05$.

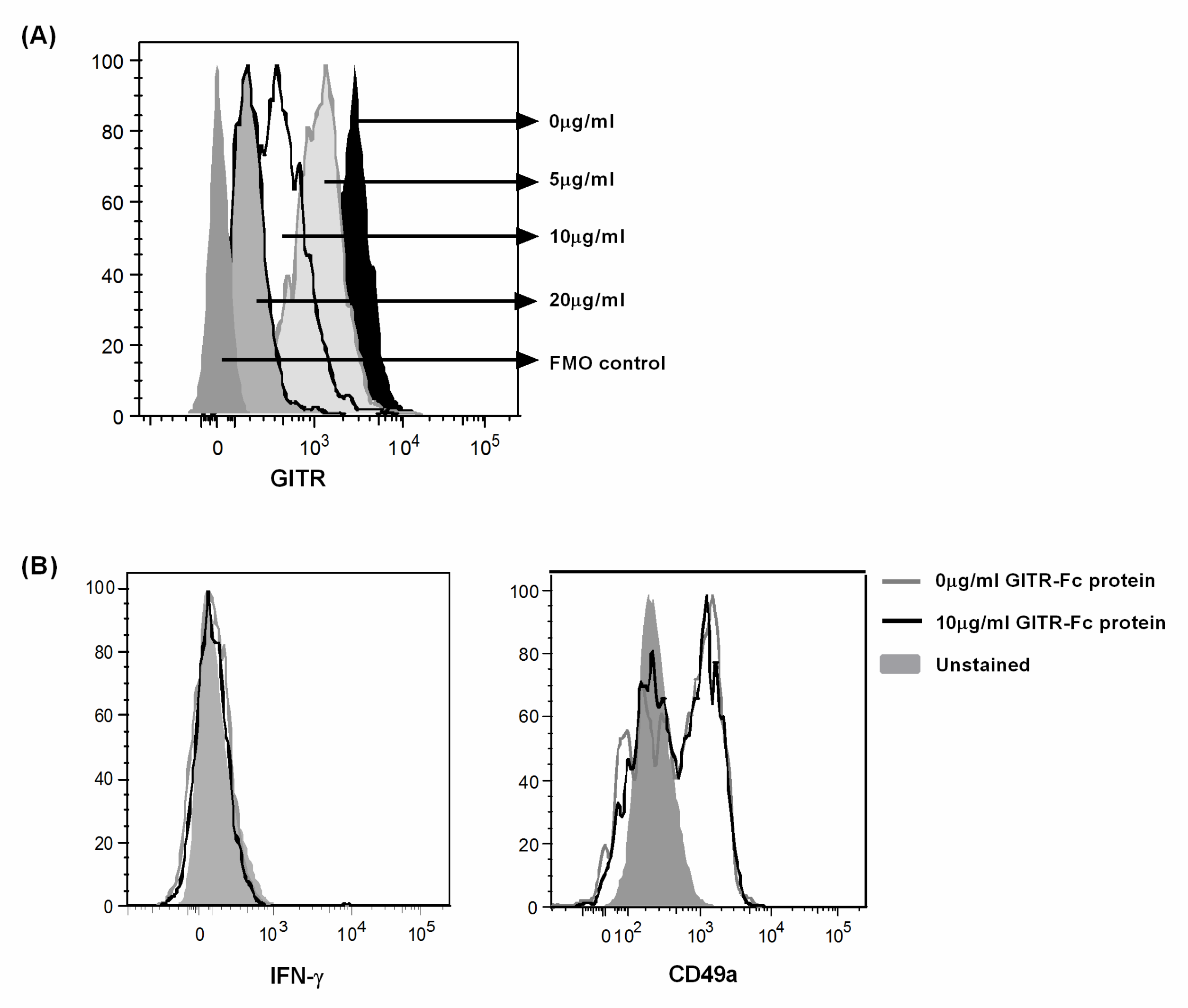

Figure S7: Modulation of GITR expression *in vitro* upon treatment with GITR-Fc chimera protein. GITR-Fc treatment was performed overnight on splenocytes (2*10^5 cells) isolated from wild-type mice utilizing the recombinant mouse GITR-Fc fusion chimera protein. Subsequently, surface and intracellular cytokine staining was performed and $CD3^-CD19^-Gr1^-$ $CD90^+CD127^+NKp46^+$ lymphocytes were analyzed by flow cytometry (n = 3-4 technical replicates). (A) Representative histograms showing GITR-Fc-induced reduction in GITR expression at the indicated concentrations. (B) Representative histograms displaying the MFI of IFN-γ and CD49a of $CD3^-CD19^-Gr1^-CD90^+CD127^+NKp46^+$ lymphocytes after overnight treatment with 10µg/ml of the GITR-Fc fusion chimera protein and GITR-expressing control (0µg/ml GITR-Fc). The presented data are representative from one out of two independent experiments.

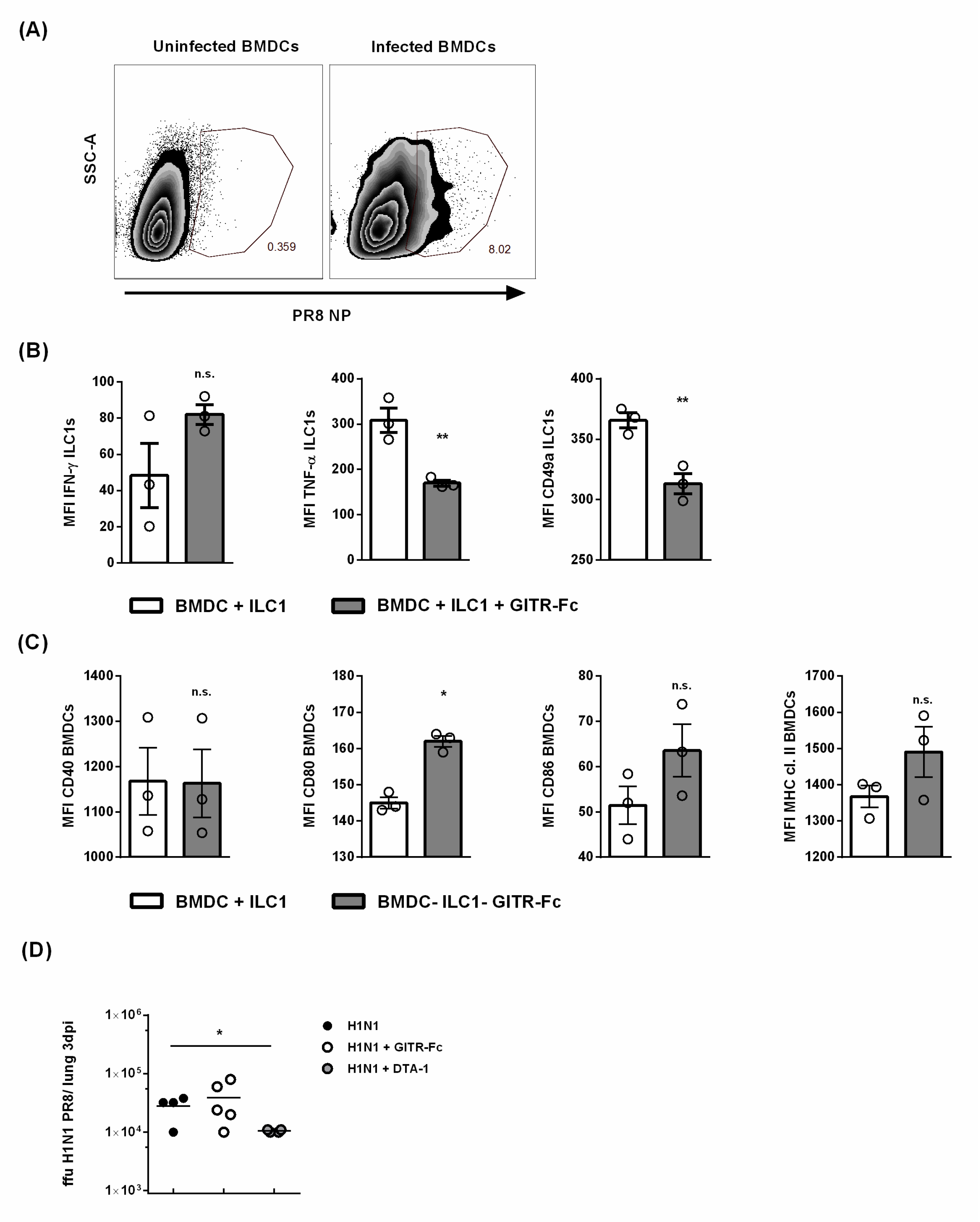

Figure S8: Impact of GITR interference on ILC1s and BMDCs. FLT-3 ligand-differentiated BMDCs were cultured overnight with *in vitro*-generated ILC1s at a 1:1 ratio. In addition, modulation of GITR expression by ILC1s was performed *in vitro* overnight using the GITR-Fc chimera protein. (A) Representative FACS plots showing the infection efficacy of BMDCs by

H1N1 PR8. (B) MFI of IFN-γ, TNF-α and CD49a by ILC1s upon coculture with BMDCs and treatment with the GITR-Fc chimera protein. (C) MFI of CD40, CD80, CD86 and MHC cl. II of BMDCs upon coculture with ILC1s and treatment with the GITR-Fc chimera protein. Bars represent mean ± SEM (n = 3-4). MFI data are representative from one out of two independent experiments. For the assessment of the viral load, wild-type mice were treated either with the DTA-1 GITR agonist (500µg/animal) or the GITR-Fc chimera protein (6.25µg/animal) i.p.. After 24 h mice were infected i.n. with $2*10^3$ ffu of the H1N1 PR8 strain (n = 4-5). (D) Scatter plots represent viral loads of infected untreated, GITR-Fc chimera protein-treated and infected and DTA-1-treated and infected mice 3 dpi as determined by foci assay and depicted as ffu per lung. Asterisks denote significant values as calculated by unpaired, two-tailed Student's t-test as compared to infected untreated groups; ****$p \leq 0.0001$; *** $p \leq 0.001$; ** $p \leq 0.01$; *$p \leq 0.05$; n.s., not significant.

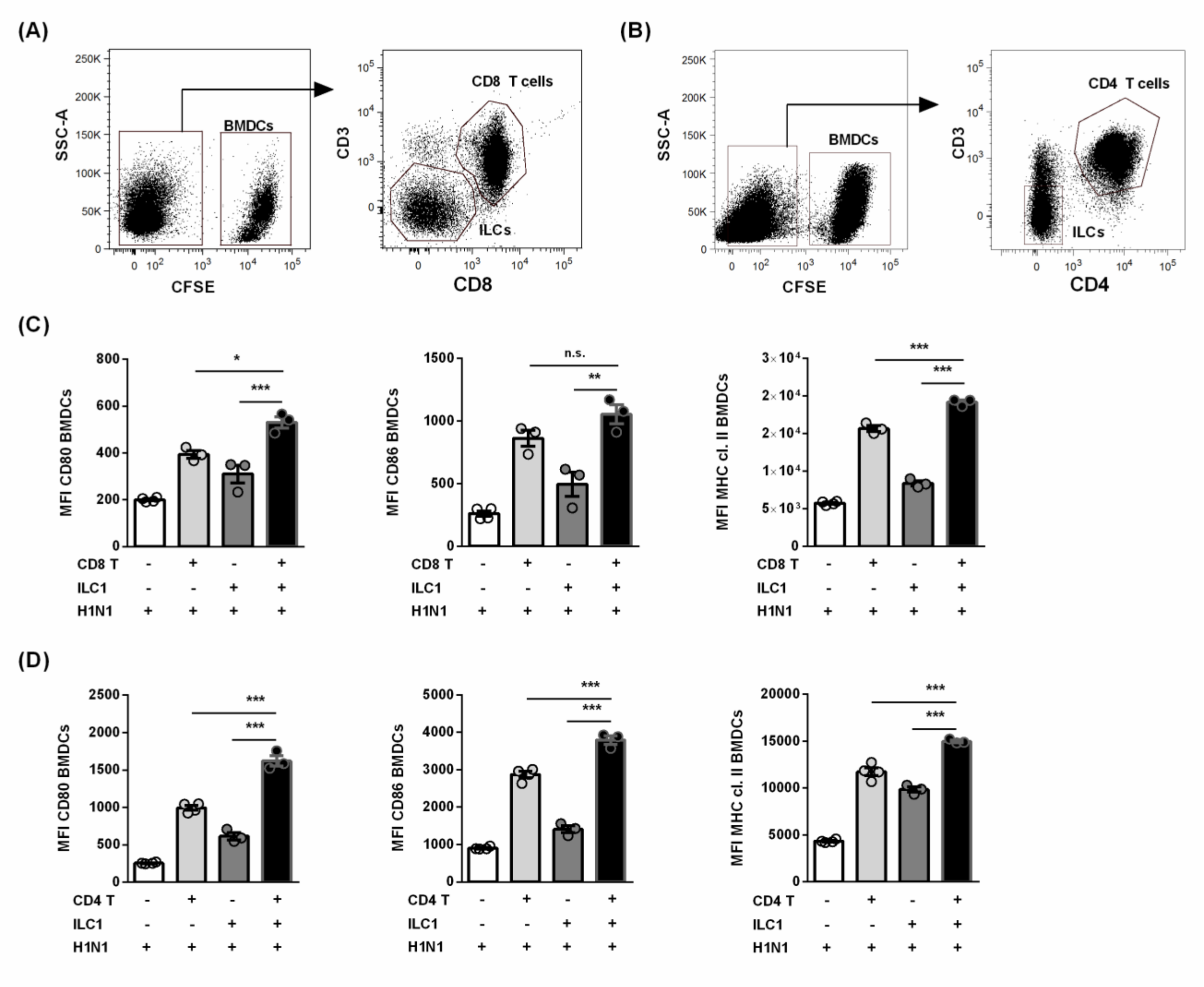

Figure S9: Cross-talk of H1N1 PR8-infected BMDCs with ILC1s and CD8/CD4 T cells. Gating strategy of *in vitro*-generated ILC1s that were cultured overnight with infected or uninfected BMDCs and (A) CD8 T or (B)CD4 T cells derived from OT I or OT II mice respectively, in different combinations at a 1:1:1 ratio. Surface markers and cytokines were assessed by flow cytometry analysis. BMDCs were identified by CFSE staining. CFSE-unlabeled cells were gated as $CD3^{-}CD8/4^{-}$ ILCs and $CD3^{+}CD8/CD4^{+}$ T cells. MFI of infected BMDCs expressing CD80, CD86 and MHC cl. II upon coculture with *in vitro*-generated ILC1s and (C) CD8 T or (D) CD4 T cells. Bars with scatter plots represent the mean ± SEM (n = 3-4). MFI data are representative from one out of two independent experiments. Asterisks denote significant values as calculated by One-way ANOVA comparing the indicated groups; ****$p \leq 0.0001$; *** $p \leq 0.001$; ** $p \leq 0.01$; *$p \leq 0.05$; n.s., not significant.

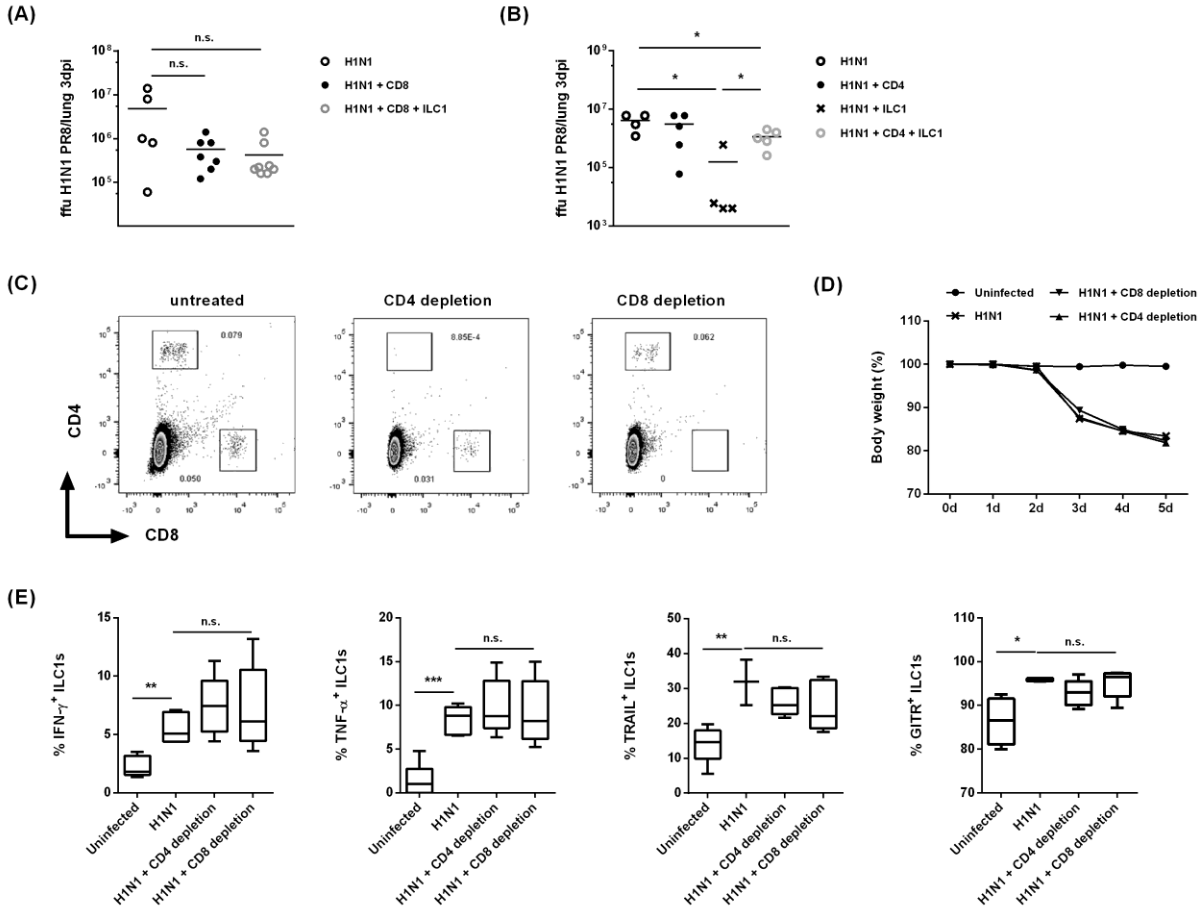

Figure S10: Cross-talk of ILC1s with CD8/CD4 T cells during H1N1 PR8 infection *in vivo*. (A) $1*10^5$ CD8 T cells and (B) $1*10^5$ CD4 T cells were adoptively transferred i.v. into RAG2$^{-/-}$γc$^{-/-}$ mice with and without an equal number of ILC1s. After 24 h mice were i.n. infected with $2*10^3$ ffu of the OVA peptide SIINFEKL-expressing (for CD8 T cells) or the aa323-393-expressing (for CD4 T cells) H1N1 PR8 influenza strain. Depicted are viral loads in the lungs of infected mice 3 dpi as determined by foci assay. Viral load data are derived from one experiment. To assess the impact on ILC1s in the absence of adaptive immune T cells, CD8 or CD4 T cells were depleted before and at day 3 of H1N1 PR8 infection of wild-type mice by i.p. administration of CD8/CD4 T cell depleting antibodies (200µg/animal). Blood samples were collected from the retro-orbital vein to confirm the depletion of CD8/CD4 T cells by flow cytometry. (C) Representative plots depicting the depletion efficacy of CD8/CD4 T cells in blood samples. Lung-derived lymphocytes of infected wild-type mice were isolated 3 dpi and incubated at 37°C in medium containing brefeldin and monensin for 3 h prior to the flow cytometry staining with regard to markers related to ILC1 activation and functionality. (D) Body weight (%) displayed for CD8/CD4 T cell depleted infected and control wild type-mice. (E) Frequencies of IFN-γ$^+$, TNF-α$^+$, TRAIL$^+$ and GITR$^+$ lung-derived ILC1s. Box plots represent the range in frequency variation with the horizontal line drawn at the mean. The represented CD8/CD4 T cell depletion data 3 dpi are derived from one experiment (n = 5-6). Asterisks denote significant values as calculated by

One-way ANOVA comparing the indicated groups; ****$p \leq 0.0001$; *** $p \leq 0.001$; ** $p \leq 0.01$; *$p \leq 0.05$; n.s., not significant.
